# Removal of Hydrogen Sulfide from Swine-Waste Biogas on a Pilot Scale Using Immobilized *Paracoccus versutus* CM1

**DOI:** 10.3390/microorganisms10112148

**Published:** 2022-10-29

**Authors:** Ladapa Kumdhitiahutsawakul, Dolruedee Jirachaisakdeacha, Uthen Kantha, Patiroop Pholchan, Pachara Sattayawat, Thararat Chitov, Yingmanee Tragoolpua, Sakunnee Bovonsombut

**Affiliations:** 1 Division of Microbiology, Department of Biology, Faculty of Science, Chiang Mai University, Chiang Mai 50200, Thailand; 2 Energy Research and Development Institute-Nakornping, Chiang Mai University, Chiang Mai 50200, Thailand; 3 Department of Environmental Engineering, Faculty of Engineering, Chiang Mai University, Chiang Mai 50200, Thailand; 4 Environmental Science Research Center (ESRC), Chiang Mai University, Chiang Mai 50200, Thailand

**Keywords:** immobilization, *Paracoccus versutus* CM1, biofilters, hydrogen sulfide, biogas, bacterial community

## Abstract

Hydrogen sulfide (H_2_S) is a toxic and corrosive component that commonly occurs in biogas. In this study, H_2_S removal from swine-waste biogas using sulfur-oxidizing *Paracoccus versutus* CM1 immobilized in porous glass (PG) and polyurethane foam (PUF) biofilters was investigated. Bacterial compositions in the biofilters were also determined using polymerase chain reaction-denaturing gradient gel electrophoresis (PCR-DGGE). The biofilters were first tested on a laboratory scale under three space velocities (SV): 20, 30, and 40 h^−1^. Within 24 h, at an SV of 20 h^−1^, PG and PUF biofilters immobilized with *P. versutus* CM1 removed 99.5% and 99.7% of H_2_S, respectively, corresponding to the elimination capacities (EC) of 83.5 and 86.2 gm^−3^ h^−1^. On a pilot scale, with the horizontal PG-*P. versutus* CM1 biofilter operated at an SV of 30 h^−1^, a removal efficiency of 99.7% and a maximum EC of 113.7 gm^−3^ h^−1^ were achieved. No reduction in methane content in the outlet biogas was observed under these conditions. The PCR-DGGE analysis revealed that *Paracoccus, Acidithiobacillus*, and *Thiomonas* were the predominant bacterial genera in the biofilters, which might play important roles in H_2_S removal. This PG–*P. versutus* CM1 biofiltration system is highly efficient for H2S removal from swine-waste biogas.

## 1. Introduction

Biogas is an important renewable energy that is produced and used worldwide for heat and electricity [1,2,3,4]. Biogas production is also an efficient waste management solution for commercial animal farms, e.g., chicken, dairy, and swine farms, and is also considered one of the solutions to tackle global warming, air pollution, and energy security [1,2,5,6,7,8]. 

Hydrogen sulfide (H_2_S) in biogas is undesirable and has become a serious issue in biogas utilization. H_2_S is a toxic and highly corrosive gas released when sulfuric acid reacts with oxygen and moisture [9]. It can cause damage to biogas equipment, generators, combustion engines, and other metal components [10]. This results in a high cost of machine maintenance. Moreover, H_2_S is a hazardous gas that affects the respiratory system [11]. The concentration and the exposure time correspond to the effects of H_2_S on health. For example, the H_2_S concentration of 2–5 ppm_v_ may cause nausea and headaches, whereas the sense of smell may be lost after 2–15 min exposure to H_2_S at 100 ppm_v_. Moreover, a concentration higher than 500 ppm_v_ can cause unconsciousness and death [12,13]. The presence of H_2_S in biogas is, thus, a concern not only for when biogas is used as an energy source but also for its toxicity to workers in biogas plants due to H_2_S exposure [12]. Therefore, it is necessary to eliminate H_2_S from the raw biogas before any use. 

Biogas has been reported to contain H_2_S in concentrations varying from 100 to 10,000 ppm_v_ but the numbers can reach up to over 30,000 ppm_v_ in some cases, depending on the composition of the feedstock used in the biogas production [14,15]. The concentrations of H_2_S in raw piggery biogas were 500–3000 ppm_v_ [16]. In Thailand, the H_2_S in the biogas produced from a pig farm was reported to be around 2000–3000 ppm_v_ [17]. Regulations were set on the level of H_2_S in biogas. For example, the amount of H_2_S in biogas must not exceed 100 ppm_v_ if the biogas is used for power generation [18,19]. For gas heating boilers and combined heat and power, most also specify a H_2_S limit to be under 1000 ppm_v_ and must be less than 1 ppm_v_ in compressed natural gas used as a transportation fuel [14].

The biological method has been regarded as a promising clean technology for reducing emissions of gases containing H_2_S, it is an effective, economical, and environmentally friendly method compared to the chemical and physical methods [20,21,22,23,24,25,26]. Biofiltration has been recognized as one of the most effective technologies for the successful removal of H_2_S from biogas using the activity of microorganisms [8,15]. Application of sulfur-oxidizing bacteria (SOB), including *Thiobacillus*, *Pseudomonas*, *Xanthomonas*, *Achromatium, Beggiatoa*, *Thiothrix*, *Rhodopseudomonas*, *Halothiobacillus* and *Acidithiobacillus* in biofiltration systems have been reported to remove H_2_S in biogas [15,20,21,22,23,24,25,26,27]. Especially, *Acidithiobacillus* sp. and *Thiobacillus* sp., which are the most commonly used genera in biological H_2_S elimination [28,29]. Members of these bacterial genera were highly effective in removing H_2_S from gas streams when compared with other species of SOB because they are highly active, easily maintained with low nutritional requirements, and capable of growing under various environmental conditions [30,31]. *Paracoccus* (formerly belonging to the genus *Thiobacillus*) [32] has been reported recently as an inoculum in a biofiltration system with a high potential to remove H_2_S from biogas [33,34,35]. In a previous study by our research group, we isolated *Paracoccus versutus* CM1 from H_2_S treatment systems and discovered that this isolate had a high efficiency in H_2_S removal during the seven-day experiment using porous glass (PG)- and polyurethane foam (PUF)-immobilized cells in biofilters [36]. The presence of *soxB*, a gene involved in thiosulfate oxidation, was also confirmed in this isolate. 

Therefore, the aims of this research were: (i) to optimize the space velocity (SV) used in a biofiltration system at a laboratory scale using *P. versutus* CM1-immobilized PG and PUF biofilters for H_2_S removal from swine-waste biogas, (ii) to investigate the performance of vertical and horizontal pilot-scale biofilters (iii) to investigate the stability of *P. versutus* CM1 and identify the major bacteria potentially involved in H_2_S removal through bacterial community analysis in both laboratory- and pilot-scale biofiltration systems using polymerase chain reaction-denaturing gradient gel electrophoresis (PCR-DGGE). 

## 2. Materials and Methods

### 2.1. Immobilization of P. versutus CM1 on PG and PUF

#### 2.1.1. Cultivation of *P. versutus* CM1 

The pure culture of *P. versutus* CM1 (accession number MN416304) was grown in thiosulfate (TS) medium (pH 7.0), which contained (per liter): 10.0 g Na_2_S_2_O_3_·5H_2_O, 1.0 g NH_4_Cl, 0.5 g MgCl_2_·6H_2_O, 0.3 g MgSO_4_·7H_2_O, 0.2 g CaCl_2_·2H_2_O, 0.2 g FeCl_3_·6H_2_O, 0.4 g KH_2_PO_4_, and 0.6 g K_2_HPO_4_. The inoculum was prepared by cultivating a single colony in 250 mL of TS liquid medium, followed by incubation at 37 °C for 72 h in a rotary shaker at 200 rpm. Colonies of *P. versutus* CM1 on TS agar were smooth, circular, and white in color. The cells were non-spore-forming, Gram-negative coccoid or short-rod-shaped with a smooth cell surface with sizes of 1.0–2.0 µm. The chemolithoautotrophic *P. versutus* CM1 was able to grow under aerobic and microaerobic conditions [36].

#### 2.1.2. Preparation of Packing Materials and Immobilization 

Porous glass (PG) and polyurethane foam (PUF) were used as the packing materials in this study. PG was prepared at the Department of Industrial Chemistry, Chiang Mai University using recycled colorless cullet provided by Kaew Sing (2000) Co., Ltd. (Khon Kaen, Thailand). PUF was obtained from Siam Nathan International Co., Ltd., (Samut Prakan, Thailand). 

PG and PUF were prepared for immobilization according to the methods described by Jirachaisakdeacha [36]. Both materials were washed with clean water. PG was added to thiosulfate liquid medium in a polycarbonate carboy culture tank (Nalgene, Rochester, NY, USA). PUF was added to a plastic container tank. These were then sterilized by autoclaving at 121 °C for 15 min. Subsequently, an inoculum (3% (*v*/*v*)) of *P. versutus* CM1 was added, followed by incubating at ambient temperatures with filtered-air aeration for 72 h to allow the growth of the bacterium. The growth of immobilized *P. versutus* CM1 cells on PG and PUF was investigated using serial dilution-spread plating on TS agar after incubating at 37 °C for 72 h, and colony forming units (CFU) per gram of immobilizing materials were calculated.

#### 2.1.3. Scanning Electron Microscope (SEM) Analysis

Scanning electron microscope was used to observe the bacterial adsorption on the materials, the cell suspension of *P. versutus* CM1 was inoculated into PG and PUF and incubated at 37 °C for 72 h. The samples were cut into smaller pieces (<1 cm) and 2.5% glutaraldehyde was added for sample fixation. The samples were then rinsed three times with 0.1 M phosphate-buffered saline (PBS), followed by an ethanol dehydration series. The samples were then completely dried, mottled, coated with gold, and observed under the Jeol JSM-5910 scanning electron microscope, hereinafter SEM, (JEOL USA, Inc., Peabody, MA, USA).

#### 2.1.4. Effect of pH and Temperature on the Immobilized *P. versutus* CM1

The effects of pH and temperature on immobilized *P. versutus* CM1 were also studied. The effects of pH were studied by soaking the immobilized materials in buffer with different pH values (4, 5, 7, and 12) at 37 °C for 5 days. In the case of temperature, the immobilized *P. versutus* CM1 on PG and PUF were incubated at temperatures of 25, 30, 35, 40, and 45 °C for 5 days. Samples of the immobilized cells were aseptically collected and subjected to a ten-fold serial dilution in 0.85% (*w*/*v*) sterile sodium chloride before being spread on a TS agar plate. *P. versutus* CM1 was incubated aerobically at 37 °C for 72 h to assess the growth. Total colony counts as CFU were also determined.

#### 2.1.5. Statistical Analysis

Mean of the triplicates and standard deviation were calculated using Microsoft Excel 2010. A one-way analysis of variance (ANOVA) was used to measure the statistical significance of the test parameters using SPSS 22.0 software (IBM SPSS Statistics for Windows, Version 22.0, IBM, SPSS Inc., Chicago, IL, USA) and Duncan’s multiple range tests. Significance was considered using *p* < 0.05.

### 2.2. Designing Laboratory- and Pilot-Scale Biofiltration Systems for H_2_S Removal

#### 2.2.1. Biofilters Set up and Operation

**Figure 1:**
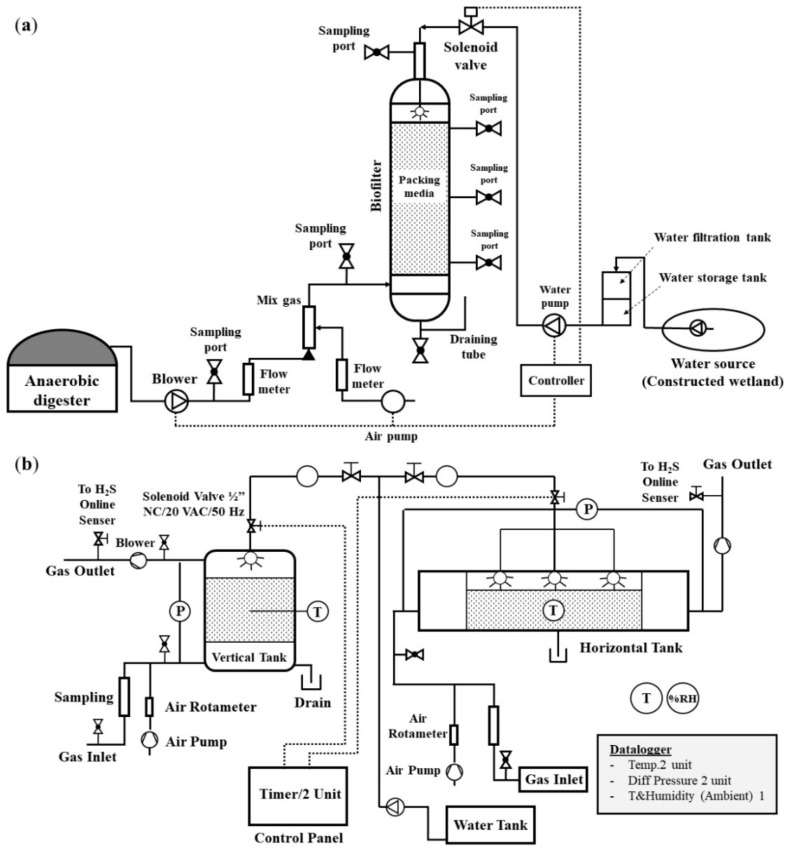
The schematic diagram of the (**a**) laboratory- and (**b**) pilot-scale biofiltration systems.

A schematic diagram of the laboratory-scale and pilot-scale biofiltration for H_2_S removal from natural biogas plant is shown in Figure 1. The operational conditions of the biofilters were presented in Table 1. The laboratory-scale biofilter was set up as previously described [24]. Each biofilter was packed by filling 4 L of PG and PUF immobilized with *P. versutus* CM1. In this study, the immobilized cells of *P. versutus* CM1 on packing materials were used for the removal of H_2_S from real biogas produced from a swine-waste digester in Sanpatong District, Chiang Mai, Thailand. The swine-waste biogas consists of methane (CH_4_) (50–70%), carbon dioxide (CO_2_) (25–36%), nitrogen (N_2_) (9–19%), oxygen (O_2_) (0.4–1.9%), and H_2_S (0.2–0.4%).

The inlet H_2_S concentrations were varied between 2000 to 3700 ppm_v_. The H_2_S was continuously fed into the biofilter until the end of the experiment. The inlet and outlet H_2_S gas concentrations were measured using a portable gas detector (Geotechnical Instruments, UK) every day. 

The laboratory-scale experiments were carried out for 60 days to evaluate the performance of the biofilter with the immobilized cells under different gas retention times. The space velocity (SV) was set at 20, 30, and 40 h^−1^ and controlled by a flow meter. The control experiments were also set up using the PG laboratory-scale biofilters at SV of 30 h^−1^ without the immobilization of *P. versutus* CM1 to confirm the activity of this bacterium. In addition, the values of gas retention time (GRT) were calculated using the following equation:

(1) $$\mathrm{Gas}\;\mathrm{retention}\;\mathrm{time}\;(\mathrm{GRT})=\frac{Vf}{Q}$$

where GRT is the residence time in seconds, ***Vf*** is the volume of the filter bed contractor, and ***Q*** is the volumetric flow rate in L/s [37]. Air was supplied into the biofilter system at 5% with the biogas stream, and water was sprayed every 30 min throughout the period of the experiment to maintain the humidity in the biofilter system. 

The pilot-scale reactors were designed based on the results obtained from the laboratory-scale experiments, as shown in Figure 1b. The system consisted of both vertical and horizontal biofilters. The vertical biofilter was made of high-density polyethylene (HDPE) with an inner diameter of 0.48 m and a height of 0.97 m. The horizontal biofilter, constructed in a rectangular shape, was made of stainless steel with a dimension of 0.30 m in width, 2.08 m in length, and a height of 0.50 m. 

The packing materials were packed into a netted bag and placed in two types of pilot-scale biofilters with an effective volume of 33.3 L and 50 L for PG and PUF, respectively. The pilot-scale biofiltration was tested for 42 days. The space velocity values for the PG and PUF biofilters were set at 30 h^−1^ and 20 h^−1^, respectively. The SV conditions were chosen for each material based on the H_2_S elimination capacity with high removal efficiency (≥98%) from the laboratory-scale test. 

#### 2.2.2. Parameter Calculation

The H_2_S removal efficiency (%RE), inlet loading rate (ILR) and the elimination capacity (EC) [38,39] were calculated using the following equations:

(2) $$H_{2}S\;\mathrm{Removal}\;\mathrm{Efficiency}\;(\%\mathrm{RE})=\frac{Cin-Cout}{Cin}\times 100$$

(3) $$\mathrm{Inlet}\;\mathrm{Loading}\;\mathrm{Rate}\;(\mathrm{ILR})=\frac{Q}{V}\times C_{in}$$

(4) $$\mathrm{Elimination}\;\mathrm{Capacity}\;(\mathrm{EC})\;=\frac{Cin-Cout}{V}\times \;Q$$

where ***C_in_*** and ***C_out_*** are the H_2_S concentrations in biogas at the inlet and outlet (g/m^3^), respectively, ***Q*** is the gas flow rate (m^3^/h), and ***V*** is the volume of packing materials (m^3^). All experiments were conducted at room temperature. Relative humidity (RH), temperature, and pH were measured using a temperature–humidity datalogger and a portable pH meter, respectively. The biogas concentration of CH_4_, CO_2_, H_2_S, O_2_, and N_2_ were measured using a portable gas detector (Geotechnical Instruments, UK).

### 2.3. Analysis of Bacterial Community in Laboratory and Pilot Biofiltrations 

#### 2.3.1. Sample Collection and DNA Extraction 

The samples of PG and PUF were collected randomly from inside the laboratory-scale biofilters that operated using SV of 30 h^−1^ and 20 h^−1^ by sterile forceps. The samples were then tested to assess the stability of *P. versutus* CM1 and to investigate the bacterial community involved in H_2_S removal. For the laboratory scale, samples were collected on day 1, 10, 20, 35, 45, and 55. While, for the pilot-scale, samples were only collected at the end of the biofiltration process; therefore, 12 samples were collected randomly from the system. The collected samples were washed aseptically with sterile distilled water and vortexed vigorously for 10 min before the washing water was obtained and centrifuged at 12,000 rpm at 4 °C for 10 min. The supernatant was discarded. All samples were prepared, and DNA was extracted according to the methods described by Kumdhitiahutsawakul [40]. The extracted DNA was resuspended in 50 µL of sterile DNase-free water and the quality of the extracted DNA was checked on a 1.0% (*w*/*v*) agarose gel. 

#### 2.3.2. Polymerase Chain Reaction (PCR) 

To amplify the bacterial 16S rRNA genes for DGGE fingerprinting, the universal primer set 341f (5′CTACGGGAGGCAGCAG3′) and 907r (5′CCGTCAATTCMTTTGAGTT T3′) were used. The GC-clamp (5′CGCCCGCCGCGCCGTCCCGCCGCCCCCG3′) was attached to the primer 341f at the 5′ end [41]. The reaction mixture for PCR contained 5 μL of 10× Taq DNA Polymerase buffer, 6 µL of 25 mM MgCl_2_, 2 µL of 99% (*v*/*v*) dimethyl sulfoxide (DMSO),2 μL of 10 mM dNTPs, 2 μL of extracted DNA, 2 μL of each primer (10 μM), 0.6 µL of 5 units of Taq polymerase (Fermentas, London, UK), and sterile deionized water (up to 50 μL). The PCR was performed using the conditions as follows: initial denaturation at 94 °C for 6 min, 30 cycles of denaturation at 94 °C for 30 s, annealing at 51 °C for 30 s, and extension at 72 °C for 30 s, followed by a final extension step at 72 °C for 10 min. 

#### 2.3.3. Denaturing Gradient Gel Electrophoresis (DGGE) and Sequencing

DGGE analysis of the PCR products was carried out and performed using the Dcode^TM^ Universal Mutation Detection System (Bio-Rad, Hercules, CA, USA). The amplified fragments were separated and analyzed on a 6% (*w*/*v*) polyacrylamide gel (prepared using 40% acrylamide/bis solution with the ratio of 37.5:1) with a denaturing gradient of 30–50%. Electrophoresis was carried out in 1× TAE buffer for 5 h at 60 °C and 130 V. After electrophoresis, the gel was stained with ethidium bromide for 20 min and visualized under UV light using the digital camera attached to the Syngene™ NuGenius Gel Documentation System (Synoptics Ltd., Cambridge, UK). 

DGGE bands on the polyacrylamide gel were excised under a UV transilluminator (Synoptics Ltd., Cambridge, UK). Each gel piece was transferred to a microtube, 50 μL of nucleic acid-free water was added to the gel, and the tube was incubated at 4 °C overnight. The aqueous part containing DNA was used as a template for PCR reamplification with the same primers that had no GC-clamp attached and PCR was performed using the same conditions as previously described. The PCR products were purified using the GFX™ PCR DNA and Gel Band Purification Kit (Amersham Pharmacia Biotech, Inc., Piscataway NJ, USA) according to the manufacturer’s instructions. The purified products were subjected to sequencing (performed by First BASE Laboratories Sdn. Bhd, Malaysia). 

#### 2.3.4. Nucleotide Sequence Accession Numbers and Phylogenetic Analysis 

All of the partial 16S rRNA gene sequences, retrieved from excised DGGE bands obtained in this study, have been deposited in GenBank with the accession numbers MN181388-MN181402, MN181408-MN181423, and MN420869-MN420915 (Tables S1–S6). All sequences were assigned in the NCBI databases as uncultured bacterium, and these sequences were then compared with the nucleotide sequences available in the GenBank database using the BLAST search (http://www.ncbi.nlm.nih.gov/BLAST (accessed on 22 November 2021)). Multiple sequences were aligned using the MEGA7 software program [42]. The phylogenetic tree analysis of samples was constructed using the neighbors-joining tree method with 1000 bootstrap replications. 

## 3. Results

### 3.1. Immobilization of P. versutus CM1 on PG and PUF

The average cell numbers of *P. versutus* CM1 immobilized in PG and PUF (day 0) for biofilter startup were in the range of 9 to 10 log CFU/g with the highest cell numbers being 9.81 and 10.85 log CFU/g for PG and PUF, respectively. 

**Figure 2:**
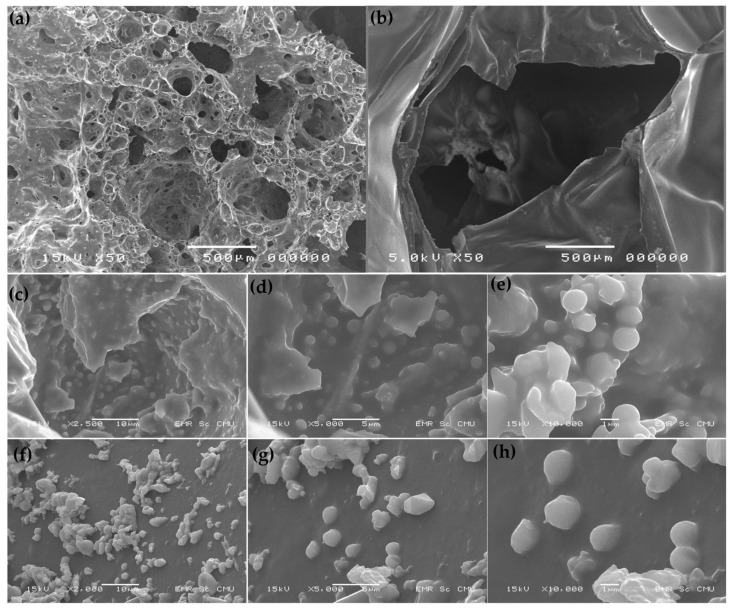
SEM images of packing materials (**a**) PG, (**b**) PUF, and immobilized *P. versutus* CM1 cells on the (**c**–**e**) PG and (**f**–**h**) PUF after 3 days of incubation.

SEM was used to assess the immobilization of the bacterium. Figure 2a,b show the structures of the packing materials, PG and PUF, respectively. After 3 days of incubation, the SEM analysis indicated that the immobilization of bacterial cells by adsorption on material surfaces was successful (Figure 2c–h). *P. versutus* CM1 was clearly observed on the surface and inside of the pores of the packing materials. 

### 3.2. Effects of pH and Temperature on the Immobilized P. versutus CM1 with PG and PUF

**Figure 3:**
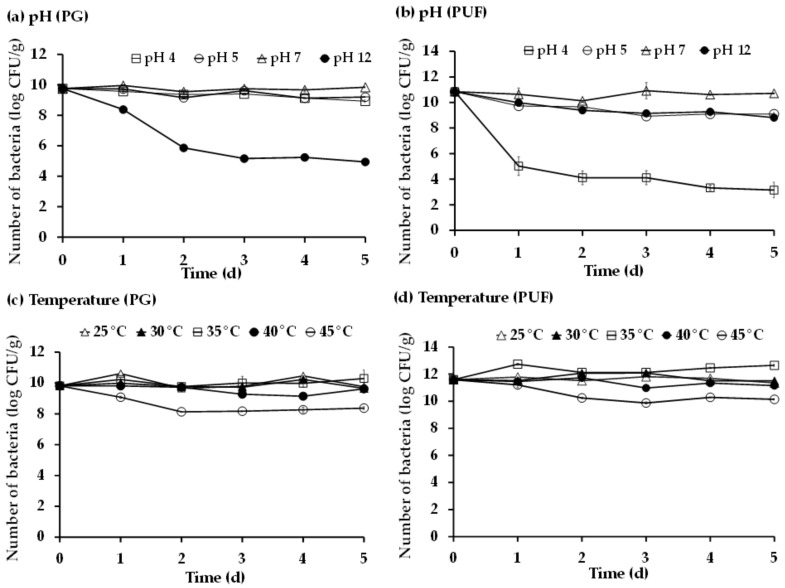
Numbers of *P. versutus* CM1 on PG and PUF under different (**a**,**b**) pH values and (**c**,**d**) temperatures.

The effects of pH and temperature on the growth of *P. versutus* CM immobilized on PG and PUF were investigated using a total colony count on thiosulfate medium. Figure 3a,b showed that the initial cells of *P. versutus* CM1 on PG and PUF were 9.81 and 10.85 log CFU/g, respectively. The numbers of *P. versutus* CM1 cells on PG at pH 4, 5, and 7 were maintained in the range of 8.93 to 9.85 log CFU/g until day 5. However, at pH 12, the number of cells rapidly decreased after 24 h (Figure 3a). On the other hand, the results from PUF showed that the number of cells rapidly decreased to 5.02 log CFU/g within 24 h at pH 4 and gradually decreased to 3.32–3.15 log CFU/g during days 4 and 5 (Figure 3b). The results indicated that the number of bacteria (log CFU/g) were statistically different at different pH values and temperatures (*p* < 0.05) (Tables S7 and S8). For example, pH 7 significantly showed higher numbers of bacteria than the other pH values. In the case of temperature, although the statistical analysis suggested that there were significant differences in cell numbers between the two packing materials at temperatures ranging from 25 to 45 °C (Figure 3c,d), they showed similar trends with an obvious decreased at 45 °C. 

### 3.3. Removal of H_2_S in Laboratory- and Pilot-Scale Biofiltration Systems 

#### 3.3.1. Effects of Gas Retention Time on H_2_S Removal in the Laboratory-Scale Experiments

**Figure 4:**
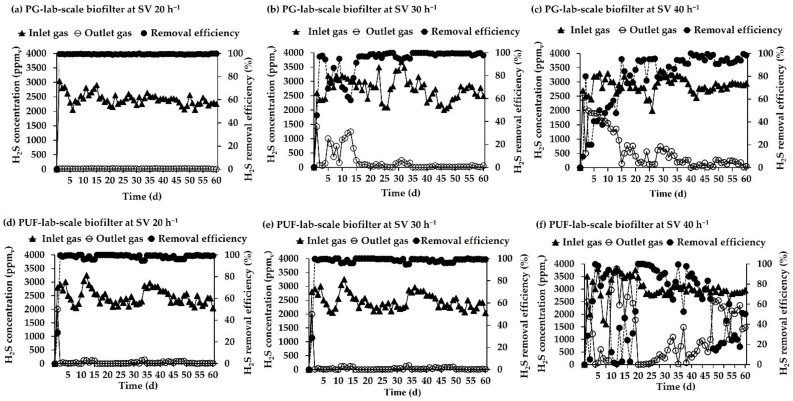
Inlet/outlet of H_2_S and efficiencies of H_2_S removal in the lab-scale biofilters packed with immobilized cells *P. versutus* CM1 on PG under different SV conditions: (**a**) 20 h^−1^, (**b**) 30 h^−1^, and (**c**) 40 h^−1^ and on PUF under different SV conditions: (**d**) 20 h^−1^, (**e**) 30 h^−1^, and (**f**) 40 h^−1^.

During the 60-day laboratory-scale operation, the biogas produced using the CMU-Channel Digester (CMU-CD) of the swine wastewater treatment plant (used as a source of H_2_S gas) fluctuated between 2005 and 3644 ppm_v_. The temperature range in the biofilters was 24 to 38 °C and the relative humidity was maintained at 89–92%. The pH from the biofilters was found within the range of 4.0 to 5.0. The performance of the laboratory-scale biofilters operated under different SVs (20, 30, and 40 h^−1^) is shown in Figure 4. 

It was found that the PG biofilter completely removed H_2_S within 24 h at the SV of 20 h^−1^ when inlet H_2_S concentrations varied between 2005 to 3644 ppm_v_ (Figure 4a). The system worked stably with a removal efficiency of 99–100% without fluctuation throughout the experiment. This indicated that the bacteria immobilized on the biofilter had sufficient activity to remove the H_2_S in biogas. Compared to the SV of 30 h^−1^, (Figure 4b), a H_2_S removal efficiency of 97% was achieved within two days, and then fluctuated before it recovered to 97% on the 16th day. After that, the stability of the biofiltration system was apparent with a high efficiency of H_2_S removal (98–100%) in the biofilter on day 35 onwards until the end of the experiment. When SV was increased to 40 h^−1^ (Figure 4c), the H_2_S removal efficiency decreased to 10–80% (days 1–14). Subsequently, the efficiency increased to 74–95% on days 15–34, and the highest removal efficiency of 100% was achieved on day 40. The system was stable during days 35–60 with a H_2_S removal efficiency of 91–100%. 

H_2_S removal in the PUF biofilter immobilized with *P. versutus* CM1 during a 60-day period is shown in Figure 4d–f. Inlet H_2_S concentrations varied between 2050 and 3244 ppm_v_. At the SV of 20 h^−1^, complete removal (100%) was accomplished within two days (Figure 4d). The H_2_S removal efficiency of 96–100% was determined throughout the experiment. The experiment at SV 30 h^−1^ (Figure 4e) showed that 100% removal was achieved within four days, but the removal efficiency dropped to 72% on the sixth day. Removal efficiencies were recovered afterward and remained at 91–100% until the end of the experiment. The biofilter at the SV of 40 h^−1^ (Figure 4f) had 100% H_2_S removal efficiency within four days, then decreased to 1–2% (day 11–12) before increasing to 98–100% during days 20–25. After that point, the biofilter performance was unstable and the efficiencies value was 14–60% until the end of the operation. 

The control biofilters without the addition of *P. versutus* CM1 were also carried out to confirm the activity of the immobilized bacterium. The results showed that the removal efficiencies were 16 and 19.2% (24 h) for PG and PUF, respectively (Figures S1 and S2). Overall, it showed that the inlet concentrations of H_2_S had little effect on the removal efficiency as the biofilter system could achieve a high removal efficiency within a short period of time. On the other hand, SV was found to have an effect on H_2_S removal. The lowest SV (20 h^−1^) resulted in a high removal efficiency when compared with 30 and 40 h^−1^ regardless of the packing materials. 

#### 3.3.2. Pilot-Scale Experiments for H_2_S Removal 

The pilot-scale experiments were carried out to evaluate H_2_S removal performance under the SVs of 30 and 20 h^−1^ for PG and PUF biofilters, respectively. The inlet H_2_S concentrations were 2290–2700 ppm_v_. The temperature in the pilot-scale biofilters was 27 to 40 °C, 88–92% RH and the pH was found within the range of 4.0 to 5.0 from the draining point.

**Figure 5:**
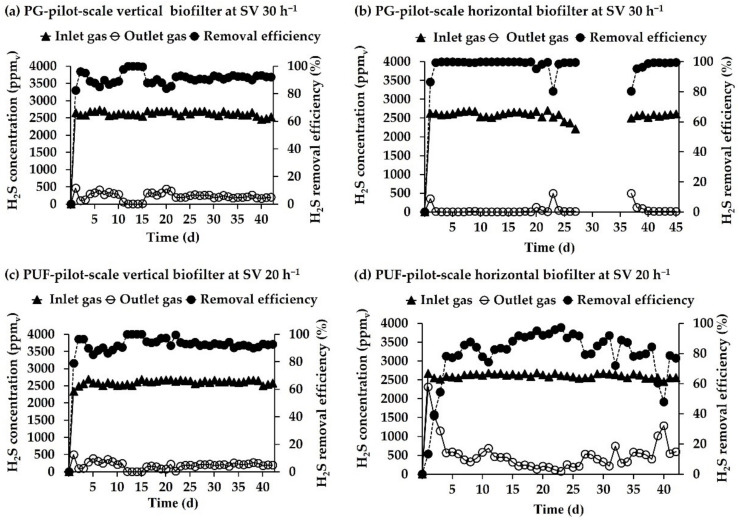
Inlet/outlet H_2_S and H_2_S removal efficiency in the pilot-scale biofilters packed with immobilized *P. versutus* CM1 cells on PG with SV of 30 h^−1^, (**a**) vertical and (**b**) horizontal positions and PUF with SV of 20 h^−1^, (**c**) vertical and (**d**) horizontal positions.

The efficiency of H_2_S removal in the biofilters packed with immobilized cells of *P. versutus* CM1 on PG was found to reach a value of 96% and 99% in the vertical and horizontal biofilters, respectively, after 2 days of operation (Figure 5a,b). The vertical biofilter packed with PG-immobilized cells showed complete removal of H_2_S within 12 days before being reduced to 88% from day 16 onwards. However, the average H_2_S removal efficiency in this biofilter was 91.9% during days 22–42 (Figure 5a). During days 3–19 of the operation, the PG-horizontal biofilter achieved a removal efficiency of 99.9% (Figure 5b). The removal efficiency dropped to 95% and 80% on days 20 and 23 of the operation before returning to 99.9% within 24 h. The missing results between days 28–36 owe to shortage of biogas generated from the anaerobic digester. After a 9-day shutdown, the performance of the horizontal PG biofilter was rapidly restored (within 1 day). The results revealed that an average removal efficiency of 99% was achieved.

As for the PUF biofilter, removal efficiencies of 96% and 88% were achieved on day 2 and 8 in the vertical and horizontal biofilters, respectively (Figure 5c,d). Thereafter, decreases in removal efficiencies in both positions of the biofilter were observed until the 12th and 16th days of the operation. The maximum H_2_S removal (100%) in a vertical PUF biofilter was achieved between days 12–15 when the average inlet H_2_S concentration was 2586 ± 77.5 ppm_v_. After that (days 16–42), the removal efficiency from the vertical biofilter was, on average, 93 ± 4.2%. H_2_S removal efficiencies in the horizontal biofilter increased to 91–97% on days 16–23 (Figure 5d). The high removal efficiency of 97% was observed on day 23 and then quickly decreased to 48–93% until the end of the operation. The average H_2_S removal efficiency in the horizontal biofilter packed with PUF was 80 ± 16.2%, which was not acceptable for a practical application as the treated biogas still contained H_2_S at a concentration higher than 250 ppm_v_, while the permitted level was ≤100 ppm_v_ for use in power generator. The collapse of the PUF material was detected, resulting in H_2_S gas being unevenly distributed in the horizontal biofilter, thus affecting the removal efficiency.

#### 3.3.3. The Performance of Laboratory- and Pilot- Scale Biofiltration on H_2_S Elimination

The H_2_S elimination capacity of biofilters on laboratory and pilot scales were compared as shown in Table 2 (Figures S3–S5). The capacity in the laboratory-scale biofilters recorded at SV of 20 and 30 h^−1^ were, on average, 66.4 and 109.0 gm^−3^ h^−1^ with high removal efficiencies of 99.4%, 98.4%, respectively. The efficiency dropped to 91.5% at the SV of 40 h^−1^ but the average H_2_S elimination capacity was still 143.1 gm^−3^ h^−1^ with the maximum H_2_S elimination capacity of 166.3 gm^−3^ h^−1^. The average elimination capacity of laboratory-scale immobilized *P. versutus* CM1 on PUF at SV of 20, 30 and 40 h^−1^ were 66.8, 100.7 and 99.3 gm^−3^ h^−^**^1^** with the removal efficiencies of 98.6, 96.8 and 62.0%, respectively. 

Interestingly, immobilization of *P. versutus* CM1 cells on PG in a horizontal pilot-scale biofilter was still possible to reach a high removal efficiency of 98.3–99.7% with a maximum H_2_S elimination capacity of 113.7 and, on average, 107.1 gm^−3^ h^−^**^1^**. Moreover, the results showed that average H_2_S elimination capacities were 68.0 and 61.3 gm^−3^ h^−1^ in vertical and horizontal pilot-scale biofilters packed with PUF, respectively. It is important to note that the elimination capacities from pilot-scale biofilters showed to be less than that of laboratory-scale biofilters. 

#### 3.3.4. CH_4_, CO_2_, N_2_ and O_2_ Concentration in Pilot-Scale Biofilters

**Figure 6:**
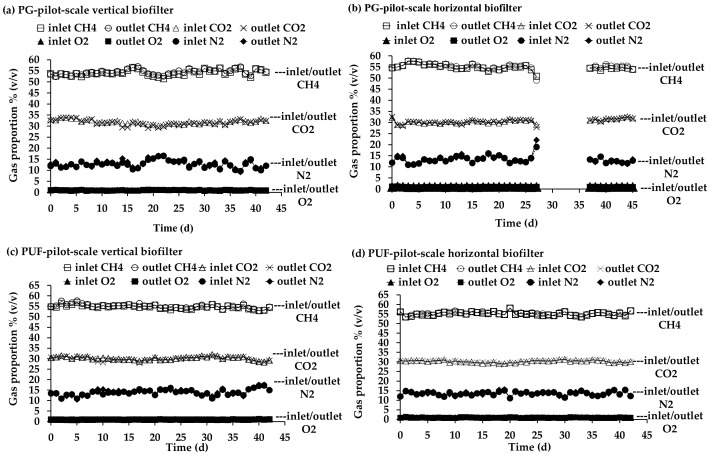
Biogas compositions in the pilot-scale biofilters packed with immobilized *P. versutus* CM1 cells on (**a**) PG vertical biofilter and (**b**) PG horizontal biofilter (**c**) PUF vertical biofilter and (**d**) PUF horizontal biofilter. The biofiltration operated at the SV of 30 and 20 h^−1^ for PG and PUF, respectively.

Concentrations of CH_4_, CO_2_, N_2_ and O_2_ in the inlet and outlet biogas in the pilot-scale biofilters were monitored to evaluate effects of the H_2_S biofiltration system on the CH_4_ and CO_2_ concentrations throughout the experiment and the resultant data from the lab-scale biofilters under the same SV is presented in Figure S6. According to the results, there was no difference between the inlet and outlet biogas from all pilot-scale biofilters (Figure 6). This indicated that the concentrations of CH_4_ and CO_2_ in the biogas were not affected by the H_2_S removal processes using immobilized *P. versutus* CM1 cells.

### 3.4. The Bacterial Community in the Laboratory and Pilot Biofiltrations

**Figure 7:**
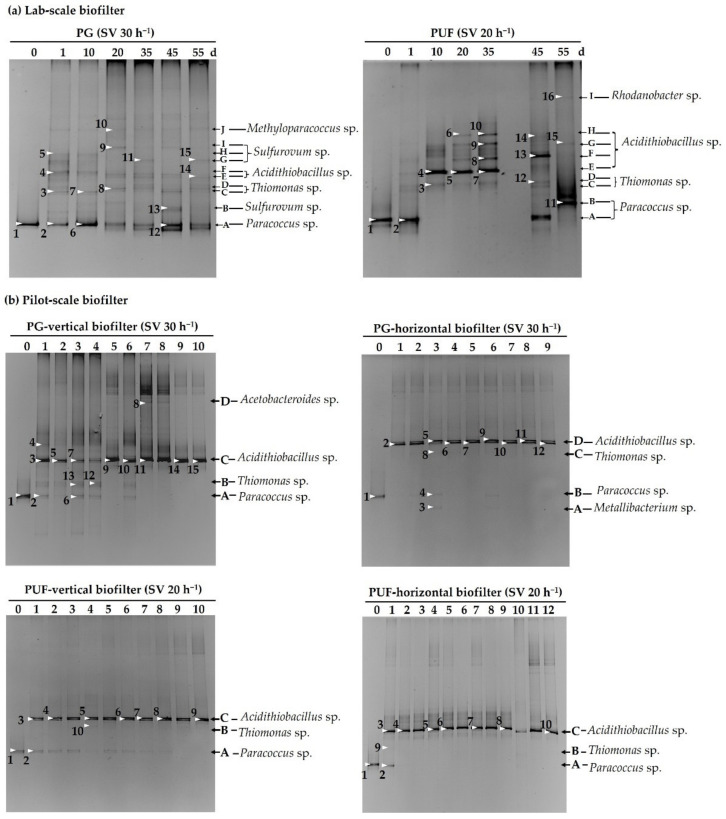
DGGE profiles of the bacteria present in the PG and PUF from laboratory-scale biofilters (**a**) and pilot-scale biofilters (**b**). The numbers on the results are from different samples collected at the end of the operation. Please note that the laboratory-scale biofiltration was carried out for 60 days; however, the DNA extraction was not successful on day 60 due to an accumulation of sulfate particles.

The stability of *P. versutus* CM1 and dominant bacteria in the biofilters were analyzed using PCR-DGGE. The DGGE profiles of the bacterial population in the biofilters are shown in Figure 7. Considering the fingerprints obtained for PG and PUF laboratory- and pilot-scale biofilters, the cells of *P. versutus* CM1 were observed on day 0 (Band 1). For PG laboratory-scale biofilter, all DGGE bands in lane A (Figure 7a, left) showed high intensity, and a DGGE band on the gel appeared in the same position as *P. versutus* CM1. It indicated that inoculated *P. versutus* CM1 was a consistently dominant bacterium that remained in these systems during the whole operation period. This indirectly provides evidence for the stability of *P. versutus* CM1 in the biofilter. The viability of *P. versutus* CM1 in the biofiltration system was also confirmed by randomly collecting the packing media and cultivating the contained microorganisms on TS agar during the system operation (Figure S6). 

Five new DGGE bands were detected after 24 h of operation time (Figure 7a), Bands 3 and 4 (including all DGGE bands in lane C-F (bands 7, 8, and 14)) were observed with high intensity in the fingerprint throughout the experiment. These bacteria were anticipated to be native bacteria from the inlet biogas and this observation may suggest that they also play an important role in H_2_S removal. 

The DGGE profile of the PUF samples in the laboratory-scale biofilter (Figure 7a, right) was similar to the results obtained from the PG samples, in which immobilized *P. versutus* CM1 was present as the dominant band (lane A, B). Interestingly, immobilized *P. versutus* CM1 was not observed in the fingerprints of days 10, 20, and 35. However, it was detected again as seen on days 45 and 55 with intense bands. In addition, Bands 4, 5 and 7 (lane E) showed higher intensities, which indicated the change of microbial structures in the biofiltration system. 

The data in Figure 7b represents the DGGE profiles of the microbes that were present in the pilot-scale biofilters at the end of the experiment. It can be seen that certain bands in the DGGE fingerprint of the PG and PUF biofilters were similar and clearly observed in all biofilters. All DGGE bands in lanes C and D from the fingerprints of the PG pilot-scale biofilter were intense and consistent with the DGGE bands observed (lane C) from the fingerprints of PUF. The DGGE profiles showed bands at the same position as band 1, which was the band of the immobilized *P. versutus* CM1. A single band similar to the immobilized *P. versutus* CM1 (lane A and lane B in the fingerprints of PG horizontal biofilter) was observed in the DGGE profiles of the pilot-scale PG and PUF, which confirmed that the original culture was still in the biofilter system and was potentially responsible for removing H_2_S.

The remarked bands were selected from the DGGE profiles and were similar (92–99% sequence identity) to 16S rRNA gene sequences of bacterial microorganisms at the genus level in database (Table 3). These results demonstrated that the bacterial community in the PG-biofilter was more diverse than that of PUF-biofilter.

A total of 31 DGGE bands collected from the laboratory-scale experiment were successfully sequenced. All had sequence similarity to the sequences of the reference organisms in the range of 92–99%. The closest related microorganisms and the percentage sequence identity are summarized in Tables S1 and S2. Fifteen sequences obtained from PG samples showed to belong to the genera *Paracoccus*, *Thiomonas*, *Acidithiobacillus, Sulfuricurvum*, *Methyloparacoccus*, and *Sulfurovum*. Sixteen sequences from the PUF samples showed that they belonged to the genera *Paracoccus*, *Thiomonas*, *Acidithiobacillus* and *Rhodanobacter* with the similarity of 94–99%. 

The bacterial communities in pilot-scale biofilters were investigated at the end of the operation. Forty-seven DGGE bands were successfully sequenced from the DGGE gel (Table 3 and Tables S3–S6)). Thirty-one out of forty-seven were grouped into the genus *Acidithiobacillus*. Nine bands were classified into the genus *Paracoccus*, which showed similarity with the inoculated *P. versutus* CM1 with 94–99% identity. Five bands were identified as *Thiomonas* sp., having 95–99% related to *T. intermida* and *T. arsenitoxydans*. Band 3 collected from the PG sample in the horizontal biofilter was found to be similar to the genus *Metallibacterium* (*M. scheffler*). Band 8 collected from the vertical biofilter was identified as *Acetobacteroides* sp. 

The phylogenetic tree of analyzed DGGE band from laboratory- and pilot-scale biofilters was constructed (Figure S8). The microorganisms in the PG sample from the laboratory-scale biofilters were composed of five major clusters in the orders *Campylobacterales*, *Rhodobacterales*, *Burkholderiales*, *Methylococcales*, and *Acidithiobacillales*, while four clusters in the order of *Rhodobacterales*, *Burkholderiales*, *Acidithiobacillales*, and *Xanthomonadales* were found in the PUF samples.

All of the bacteria in the pilot-scale biofilters belonged to Phyla α- and γ-Proteobacteria comprising six orders as follows: *Acidithiobacillales*, *Campylobacterales*, *Rhodobacterales*, *Burkholderiales*, *Methylococcales*, and *Bacteroidales*. Even though the results showed low diversity of the bacterial community, all of these genera in the orders *Acidithiobacillales*, *Campylobacterales*, *Rhodobacterales*, and *Burkholderiales* are highly functional bacteria in the H_2_S removal system. 

## 4. Discussion

Bacterial strains and support materials used for bacterial immobilization for a biofiltration system are important factors for high performance of the H_2_S removal process. Bacterial strains from natural habitats, which can tolerate high pH and salinity [43], with low nutrient requirement, low biomass accumulation, and high resistance to fluctuations in pH, temperature, moisture, pollution load, and O_2_ are desirable [44]. In our previous work, *Paracoccus versutus* CM1 isolated from an H_2_S treatment system of a biogas plant had shown 100% H_2_S removal efficiency within 24 h in a short-period (168 h) experiment [36]. Therefore, we used this strain immobilized in biofilters for H_2_S removal in both laboratory- and pilot-scale experiments over extended time periods. *P. versutus* CM1 is a sulfur-oxidizing bacterium, which can metabolize thiosulfate or sulfide into sulfate. This strain had a considerable tolerance to a wide range of pH, which was pH 4–7 when immobilized in PG and pH 5–12 when immobilized in PUF (Figure 3). 

Considering general pH values of swine-waste (pH 7.3 ± 0.5), the immobilized cells of the CM1 strain in both support materials, PG and PUF, could be retained in sufficient cell numbers (log ≥ 7 CFU/g) to rapidly start the removal of H_2_S from biogas [38,45]. The *P. versutus* CM1 strain could also be retained in high numbers in both materials in a wide range of temperatures (25 °C to 40 °C) (Figure 3c,d) making it suitable for actual operation conditions. One additional advantage of immobilization is that the immobilized bacterial cells can effectively avoid the loss of biomass and possess greater stability compared to suspended cells in sulfide in a removal reactor [46]. PG and PUF appeared to be suitable support materials for *P. versutus* CM1, as previously discovered [36], and this was confirmed by the high number of immobilized cells and the SEM analytical results.

The laboratory-scale *P. versutus* CM1 biofilters demonstrated that high H_2_S removal efficiencies (98–100%) were maintained throughout the 60-day H_2_S biofiltration process with the biofilter packed with PG when operated at SVs of 20 h^−1^ and 30 h^−1^. A higher SV (30 h^−1^) was then chosen for the PG biofilter in the pilot-scale operation. For the PUF biofilter, this level of H_2_S removal was achieved at SV of 20 h^−1^. So, it is clear that the biofilter performance was influenced by space velocity or gas retention time (GRT). The removal efficiencies were found to progressively decreased with an increase in gas flow rates (SV of 40 h^−1^, Figure 4), whereas a high removal efficiency was achieved at the lowest gas flow rates (SV of 20 h^−1^). This study was consistent with a previous report from Ho who observed a decrease in H_2_S removal efficiency from 82% to 15% when the GRT was decreased from 4 min to 1 min in laboratory-scale H_2_S elimination using immobilized *A. ferrooxidans* CP9 [47]. Other researchers also observed decreases in H_2_S removal efficiencies under shorter gas retention times. Alinezhad et al. [37] used biofilters for the H_2_S removal from a municipal wastewater treatment plant, and the removal efficiency was reduced from 98 to 92% when the GRT was changed from 20 s to 15 s. In another study, the removal efficiencies were found to be 98.7, 96.9, 86.3, and 72.8% at the GRTs of 20, 15, 10, and 5 min, respectively [39]. Solcia et al. [48] showed that the removal efficiency was constant (98.8 ± 0.30%) when the GRT was 16 s or longer, but a decrease in the GRT from 16 to 9 s led to a corresponding decrease in removal efficiency from 98.2 to 89.6%. In addition, Chen et al. [49] reported that the H_2_S removal efficiency increased from 96% to nearly 100% when the retention time was increased from 53 s to 79 s. Overall, it is advised that high H_2_S concentrations require an elevated contact time between H_2_S and the microorganisms on the packing media for efficient H_2_S removal [50]. 

It is also important to keep the biogas flow rate and inlet H_2_S concentration relatively stable for the operation of an industrial-scale biogas treatment plant [10]. 

The optimum conditions found for the PG and PUF biofilters operated at the laboratory scale provided the basis for the pilot-scale operation. The operation of the H_2_S removal system with immobilized *P. versutus* CM1 on PG material was most efficient with the horizontal biofilter operated with an SV of 30 h^−1^, giving the highest H_2_S removal efficiency and elimination capacity. A high H_2_S removal efficiency (>98%) was achieved rapidly (within 24 h) after reactor startup. The maximum EC value observed in the pilot-scale PG-*P. versutus* CM1 biofilter, operated at an SV of 30 h^−1^, was 113.7 g·m^−3^·h^−1^ (or an average EC value of 107.1 g·m^−3^·h^−1^ at the steady state (Table 2)). This value is comparable to the EC of another biofilter with another strain of *P. versutus* [36], and higher than the recorded ECs obtained from sludge and biofilters with other predominant bacterial species [39,49,51,52,53,54,55,56]. This may indicate the great potential of *P. versutus* in H_2_S removal. The maximum EC obtained from immobilized *P. versutus* CM1 was higher in the PG biofilter than in the PUF biofilter, in both vertical and horizontal positions (Table 2). It is important to mention that in these aforementioned studies, H_2_S removal was investigated using a synthetic gas, whereas in this study, a real biogas generated from a swine wastewater treatment plant was used, which should reflect how the system would work in actual situations. It is also important to note that the initial H_2_S concentration used in the biofilter startup (>2000 ppm_v_) was higher than in a previous system (100 ppm_v_) [35], in which the same bacterial species was used. Moreover, though the void ratio of the reactor packed with PG and PUF was relatively low, there was no clogging problem in the laboratory-scale and pilot-scale biofilters throughout the experimental period. 

The structure and dynamics of microbial communities in the biofilter systems were investigated. It was expected that this would provide a better understanding of the relationship between microbial diversity and biofilter performance [57]. In this study, PCR-DGGE was used to determine bacterial distribution and community structure based on the 16S rRNA gene sequence. [23,58,59,60,61,62]. The results from the DGGE analysis revealed the presence of *Paracoccus* in the PG and PUF biofilters under long-term operations. The successful operation of any waste gas treatment system is related to the selection of a proper inoculum [63]. In a sulfide removal system, *Paracoccus* sp. was reported to be a predominant bacterium that harbored *soxB* genes involved in the bio-oxidation of inorganic reduced sulfur [64]. It has also been used as a biocatalyst for simultaneous removal of ammonium, nitrite, and nitrate [65,66]. Moreover, the use of biofilms produced by a single bacterial culture as an inoculum for biofilters has been proposed to improve the H_2_S removal efficiency by reducing the startup periods [35,55].

From our findings, the major bacteria identified during and after biofilter operation were sulfur-oxidizing bacteria in the phylum Proteobacteria, which have been known to play an important role in H_2_S degradation [67]. 

The bacteria found in the biofilters packed with PG that were related to sulfide degradation include *Paracoccus*, *Thiomonas*, *Acidithiobacillus*, *Sulfuricurvum*, *Methyloparacoccus*, and *Sulfurovum* [27,68,69]. Some chemoautotrophic genera such as *Sulfurovum, Sulfuricurvum*, and *Paracoccus* are known as excellent H_2_S removing microorganisms and, are often detected after the start of a bioreactor [69,70]. *Methyloparacoccus*, a methanotrophic bacterium, was poorly observed in the laboratory-scale PG sample, and methane oxidation activities were not found in our biofiltration system.

In addition, the bacteria identified as *Paracoccus, Thiomonas, Acidithiobacillus*, and *Rhodanobacter* were found in the laboratory-scale PUF samples. Interestingly, fewer genera, including *Paracoccus, Thiomonas*, and *Acidithiobacillus* were found on both packing media in the pilot-scale biofilters, suggesting their important role in H_2_S removal in the biofilter systems. 

Although the pilot-scale biofiltration systems were inoculated with *Paracoccus versutus* CM1, *Acidithiobacillus* sp. became the predominant bacterial species at the end of the biofiltration period. This might be due to the acidic conditions developed in the biofilters. This phenomenon was similarly observed in a previous study [71], in which *Hyphomicrobium* sp., the initial inoculum in biotricking filters, was no longer the predominant species after 60 days of operation. Moreover, Charnnok [17] found *Acidithiobacillus* sp. to be a major bacterial group in a biofilter for treating H_2_S from biogas under acidic conditions (pH 4.0–5.0). In another study, the microbial community analysis of biofilm samples showed the presence of *A. caldus*, which was predominant in all H_2_S biofilters [39]. Nevertheless, *Paracoccus* was still present in a significant proportion in the biofilter systems, and the control comparison (Figures S1 and S2) confirmed its role in removing H_2_S, especially at the start of the biofiltration process. 

## 5. Conclusions 

The porous glass (PG) biofilter immobilized with *Paracoccus versutus* CM1 was successfully adapted for pilot-scale removal of H_2_S from swine-waste biogas. The operating conditions in this study (immobilized *P. versutus* CM1 on PG with an inoculum load of approximately 10 log CFU/g, with an H_2_S inlet in the range of 2290–2700 ppm_v_, operated in the horizontal biofilter position under the SV of 30 h^−1^ at temperatures between 27 and 40 °C) are applicable for H_2_S removal from biogas from swine wastewater treatment plants. *Paracoccus* remained in the system until the end of the biofiltration process. The high efficiency (≥98%) of H_2_S removal from biogas was achieved while maintaining a high CH_4_ concentration. The analysis of bacterial community revealed that it mainly consisted of sulfur-oxidizing bacteria, indicating their association with the activity to eliminate H_2_S in the biofiltration system.

## Figures and Tables

**Table 1:** Operating parameters of the laboratory- and pilot-scale biofilters.

Parameters of Operation	Biofilters			
	Laboratory-Scale	Pilot-Scale		
		Vertical	Horizontal	
Biogas flow rate	0.079, 0.118, 0.157	1.0	1.0	m^3^/h
Air flow rate	0.004, 0.006, 0.008	0.05	0.05	m^3^/h
Reactor size	0.1 × 0.6	0.48 × 0.97	0.3 × 2.0 × 0.5	m
Column volume	0.005	0.175	0.300	m^3^
Packing volume	0.004 (PG, PUF)	0.033 (PG)	0.033 (PG)	m^3^
		0.050 (PUF)	0.050 (PUF)	m3
Space velocity (SV)	20, 30, 40	30 (PG)	30 (PG)	h^−1^
		20 (PUF)	20 (PUF)	h^−1^
Gas retention time	180, 120, 90	120 (PG),	120 (PG),	s
(GRT)		180 (PUF)	180 (PUF)	s
CH_4_ content,	58–61	58–60	58–60	% (*v*/*v*)
CO_2_ content	27–29	26–28	26–28	% (*v*/*v*)
H_2_S content (gas)	2005–3644	2290–2700	2290–2700	ppm_v_
H_2_S content (gas)	2.78–5.01	2.78–5.01	2.78–5.01	g/m^3^
Temperature	24–38	28–38	28–38	°C
Relative Humidity (RH)	89–92	78–91	78–91	% RH

**Table 2:** Comparison of the H_2_S elimination capacity in the laboratory- and pilot-scale biofilters packed with immobilized *P. versutus* CM1 cells on PG and PUF.

Biofilter/Packing Materials	SV (h^−1^)	EC Max (gm^−3^ h^−1^)	RE Max (%)	EC Average (gm^−3^ h^−1^)	RE Average (%)	Days of Average
Lab-scale/PG	40	166.3	94.3	143.1	91.5	18-60
	30	141.6	93.5	109.0	98.4 **	16-60
	20	83.5	99.5	66.4	99.4 **	1-60
Lab- scale/PUF	40	204.1	98.2	99.3	62.0	26-60
	30	135.9	100	100.7	96.8	16-60
	20	86.2	96.5	66.8	98.6 **	2-60
Pilot-scale/PG	30	110.0	100	100.6	90.9	16-42
/PUF (vertical)	20	74.5	100	68.0	93.1	16-42
Pilot-scale/PG	30	113.7	99.7	107.1	98.3 **	1-27
				107.0 *	98.5 *	38-45 *
/PUF (horizontal)	20	71.34	95.8	61.3	84.4	16-42

* The second phase was operated after biogas shutdown, ** high removal efficiency (≥98% removal efficiency (RE)) in the laboratory- and pilot-scale experiments.

**Table 3:** Identification of nucleotide sequences obtained from DGGE bands.

Microorganism Closest Relative to Nucleotide Sequences Excise from DGGE Profiles	Laboratory-Scale		Pilot-Scale Biofilter			
	Biofilter		Vertical		Horizontal	
	PG	PUF	PG	PUF	PG	PUF
*Paracoccus* sp.	1, 2, 6, 12	1, 2, 11	1, 2, 6	1, 2	1, 4	1, 2
*Thiomonas* sp.	3, 7, 8	3, 12	12, 13	10	8	9
*Acidithiobacillus* sp.	4, 14	4, 5, 6, 7, 8, 9, 10, 13, 14, 15	3, 4, 5, 7, 9, 10, 11, 14, 15	3, 4, 5, 6, 7, 8, 9	2, 5, 6, 7, 9, 10, 11, 12	3, 4, 5, 6, 7, 8, 10
*Sulfuricurvum* sp.	5					
*Sulfurovum* sp.	9, 11, 13, 15					
*Methyloparacoccus* sp.	10					
*Rhodanobacter* sp.		16				
*Acetobacteroides* sp.			8			
*Metallibacterium* sp.					3	

## Data Availability

Data contained within the article and Supplementary Materials.

## Supplementary Materials

The following supporting information can be downloaded at: https://www.mdpi.com/article/10.3390/microorganisms10112148/s1, Tables S1–S6. Closest related microorganisms to nucleotide sequences excised from DGGE profiles from PG-laboratory scale biofilter compared with the NCBI nucleotide GenBank database. Table S7. The statistical analysis was performed using one-way analysis of variance (ANOVA) and Duncan’s multiple range tests (pH). Table S8. The statistical analysis was performed using one-way analysis of variance (ANOVA) and Duncan’s multiple range tests (temperature). Figure S1: Comparison of biofilter performances between the PG laboratory-scale control biofilter without the immobilization of *Paracoccus versutus* CM1 and the PG laboratory-scale biofilter with *P. versutus* CM1; Figure S2: Comparison of biofilter performances between the PUF laboratory-scale control biofilter without the immobilization of *Paracoccus versutus* CM1 and the PUF laboratory-scale biofilter with *P. versutus* CM1; Figure S3: Laboratory-scale H_2_S elimination in various SV in PG biofilters; Figure S4: Laboratory-scale H_2_S elimination in various SV in PUF biofilters; Figure S5: Pilot-scale H_2_S elimination in PG and PUF of vertical and horizontal biofilters; Figure S6: Biogas compositions in the laboratory-scale biofilters packed with immobilized *P. versutus* CM1 cells. (a) PG biofilter and (b) PUF biofilter. The biofiltration was operated at the SV of 30 and 20 h^−1^, respectively; Figure S7: Colonies of *P. versutus* CM1 on TSA medium which collected from packing materials during biofilter operation; Figure S8: The phylogenetic tree constructed from partial 16S rRNA sequences of bacteria indicates the major bacterial taxa present in the PG and PUF of (a) laboratory-scale biofilters, and (b) pilot-scale biofilter using a neighbor-joining algorithm with 1000 bootstrapping.
