# Supplementary material for: Removal of Hydrogen Sulfide from Swine-Waste Biogas on a Pilot Scale Using Immobilized Paracoccus versutus CM1
Source: Microorganisms. 2022 Oct 29;10(11):2148. doi: 10.3390/microorganisms10112148 (PMC9693040; doi:10.3390/microorganisms10112148)
Supplement: Supplementary file 1 [file microorganisms-10-02148-s001.zip › microorganisms-1981999-supplementary.pdf]

microorganisms

MDPI

Supplementary information

Table S1. Closest related microorganisms to nucleotide sequences excised from DGGE profiles from PG-laboratory scale biofilter compared with the NCBI nucleotide GenBank database

DGGE band	GenBank accession numbers	Closest related microorganisms to nucleotide sequences excised from DGGE profiles compared with GenBank database	
		**Standard databases (nr etc.)**	**rRNA/ITS databases (16 S rRNA)**
1PG	MN181388	*Paracoccus versutus (99.61%), Paracoccus sp. strain CM1 (99.6%)*	*Paracoccus versutus (99.61%)*
2PG	MN181389	*Paracoccus versutus (98.86%)*	*Paracoccus versutus (98.86%)*
3PG	MN181390	*Thiomonas sp. (99.81%)*	*Thiomonas arsenitoxydans (99.81), Thiomonas intermedia (99.24%)*
4PG	MN181391	*Acidithiobacillus sp. (96.92%)*	*Acidithiobacillus caldus (96.15%)*
5PG	MN181392	*Uncultured Sulfuricurvum sp. (99.15%)*	*Sulfuricurvum kujiense (98.87%)*
6PG	MN181393	*Paracoccus versutus (96.59%)*	*Paracoccus versutus (96.59%)*
7PG	MN181394	*Thiomonas sp. (99.62%)*	*Thiomonas arsenitoxydans (99.62%), Thiomonas intermedia (99.04%)*
8PG	MN181395	*Thiomonas sp. (95.85%)*	*Thiomonas arsenitoxydans (95.85%), Thiomonas intermedia (95.65%)*
9PG	MN181396	*Sulfurovum riftiae (92.40%)*	*Sulfurovum riftiae (92.40%)*
10PG	MN181397	*Methyloparacoccus murrellii (92.20%)*	*Methyloparacoccus murrellii (92.20%)*
11PG	MN181398	*Sulfurovum riftiae (92.61%)*	*Sulfurovum riftiae (92.61%)*
12PG	MN181399	*Paracoccus versutus (98.85%)*	*Paracoccus versutus (98.85%)*
13PG	MN181400	*Sulfurovum riftiae (92.54%)*	*Sulfurovum riftiae (92.54%)*
14PG	MN181401	*Acidithiobacillus sp. (98.08%)*	*Acidithiobacillus caldus (97.7%)*
15PG	MN181402	*Sulfurovum riftiae (97.26%), Sulfurovum lithotrophicum (96.58%),*	*Sulfurovum riftiae (97.26%)*

Table S2. Closest related microorganisms to nucleotide sequences excised from DGGE profiles from PUF-laboratory scale biofilter compared with the NCBI nucleotide GenBank database

DGGE band	Accession numbers	Closest related microorganisms to nucleotide sequences excised from DGGE profiles compared with GenBank database	
		**Standard databases (nr etc.)**	**rRNA/ITS databases (16 S rRNA)**
1PUF	MN181408	*Paracoccus versutus (99.6%), Paracoccus sp. strain CM1 (99.4%)*	*Paracoccus versutus (99.6%)*
2PUF	MN181409	*Paracoccus versutus (99.3%)*	*Paracoccus versutus (99.3%)*
3PUF	MN181410	*Thiomonas sp. (99.8%)*	*Thiomonas arsenitoxydans (99.8%), Thiomonas intermedia (99.2%)*
4PUF	MN181411	*Acidithiobacillus thiooxidans (98.0%) Acidithiobacillus albertensis*	*Acidithiobacillus thiooxidans (98.0%)*
5PUF	MN181412	*Acidithiobacillus thiooxidans (98.6%)*	*Acidithiobacillus thiooxidans (98.6%)*
6PUF	MN181413	*Acidithiobacillus thiooxidans (99.6%)*	*Acidithiobacillus thiooxidans (99.6%)*
7PUF	MN181414	*Acidithiobacillus thiooxidans (99.2%)*	*Acidithiobacillus thiooxidans (99.2%)*
8PUF	MN181415	*Acidithiobacillus thiooxidans (99.2%)*	*Acidithiobacillus thiooxidans (99.2%)*
9PUF	MN181416	*Acidithiobacillus thiooxidans (99.8%), Acidithiobacillus albertensis (99.8%)*	*Acidithiobacillus thiooxidans (99.8%), Acidithiobacillus thiooxidans (99.8%)*
10PUF	MN181417	*Acidithiobacillus thiooxidans (99.8%)*	*Acidithiobacillus thiooxidans (99.6%)*
11PUF	MN181418	*Paracoccus versutus (94.62%)*	*Paracoccus versutus (94.2%)*
12PUF	MN181419	*Thiomonas sp. (99.6%)*	*Thiomonas arsenitoxydans (99.6%), Thiomonas intermedia (99.0%)*
13PUF	MN181420	*Acidithiobacillus thiooxidans (99.8%)*	*Acidithiobacillus thiooxidans (99.6%)*
14PUF	MN181421	*Acidithiobacillus thiooxidans (99.8%)*	*Acidithiobacillus thiooxidans (99.6%)*
15PUF	MN181422	*Acidithiobacillus thiooxidans (99.6%)*	*Acidithiobacillus thiooxidans (98.6%)*
16PUF	MN181423	*Rhodanobacter glycinis (98.6%)*	*Rhodanobacter glycinis (98.3%)*

Table S3. Closest related microorganisms to nucleotide sequences excised from DGGE profiles from PG-vertical pilot-scale biofilter compared with the NCBI nucleotide GenBank database

DGGE band	GenBank accession numbers	Closest related microorganisms to nucleotide sequences excised from DGGE profiles compared with GenBank database	
		**Standard databases (nr etc.)**	**rRNA/ITS databases (16 S rRNA)**
1PG_VP1	MN420882	*Paracoccus versutus (99.8%), Paracoccus sp. strain CM1 (99.8%)*	*Paracoccus versutus (99.8%)*
2PG_VP2	MN181883	*Paracoccus versutus (98.2%), Paracoccus sp. strain CM1 (98.2%)*	*Paracoccus versutus (98.2%)*
3PG_VP3	MN181884	*Acidithiobacillus sp. strain V1 (MT009439) (99.47%)*	*Acidithiobacillus caldus strain KU 1 (96.49%)*
4PG_VP4	MN181885	*Uncultured epsilon proteobacterium clone H2OTU12 (KM016254) (97.8%), Sulfuricurvum kujiense (97.8%)*	*Sulfuricurvum kujiense (97.8%)*
5PG_VP5	MN181886	*Acidithiobacillus sp. strain V1 (99.4%)*	*Acidithiobacillus caldus strain KU 1 (96.49%)*
6PG_VP6	MN181887	*Paracoccus versutus strain NLB7 (99.2%)*	*Paracoccus versutus strain ATCC 25364 (99.2%)*
7PG_VP7	MN181888	*Acidithiobacillus sp. strain V1 (97.8%)*	*Acidithiobacillus caldus strain KU (95.0%)*
8PG_VP8	MN181889	*Acetobacteroides hydrogenigenes (98.1%)*	*Acetobacteroides hydrogenigenes strain RL-C (98.1%)*
9PG_VP9	MN181890	*Uncultured bacterium clone AS3cl15 (KU249580) (98.94%), Acidithiobacillus thiooxidans (98.7%)*	*Acidithiobacillus thiooxidans (98.7%)*
10PG_VP10	MN181891	*Uncultured bacterium clone AS3cl15 (KU249580) (98.25%), Acidithiobacillus thiooxidans (98.08%)*	*Acidithiobacillus thiooxidans (98.08%)*
11PG_VP11	MN181892	*Uncultured bacterium clone AS3cl15 (KU249580) (98.2%), Acidithiobacillus thiooxidans (98.06%)*	*Acidithiobacillus thiooxidans (98.06%)*
12PG_VP12	MN181893	*Thiomonas sp. (99.6%)*	*Thiomonas arsenitoxydans (99.6%), Thiomonas intermedia (99.1%)*
13PG_VP13	MN181894	*Thiomonas intermedia (98.54%), Thiomonas sp. (98.54%)*	*Thiomonas intermedia (98.54%)*
14PG_VP14	MN181895	*Uncultured bacterium clone AS3cl15 (KU249580) (98.07%), Acidithiobacillus thiooxidans (97.89%)*	*Acidithiobacillus thiooxidans (97.89%)*
15PG_VP15	MN181896	*Thiomonas sp. CB2 (99.6%)*	*Thiomonas arsenitoxydans strain 3As (99.64%), Thiomonas intermedia strain ATCC 15466 (99.1%)*

Table S4. Closest related microorganisms to nucleotide sequences excised from DGGE profiles from PUF-vertical pilot-scale biofilter compared with the NCBI nucleotide GenBank database

DGGE band	GenBank accession numbers	Closest related microorganisms to nucleotide sequences excised from DGGE profiles compared with GenBank database	
		**Standard databases (nr etc.)**	**rRNA/ITS databases (16 S rRNA)**
1PUF_VP1	MN420906	*Paracoccus versutus strain NLB7 (99.4%), Paracoccus sp. strain CM1 (98.4%)*	*Paracoccus versutus strain ATCC 25364 (99.4%)*
2PUF_VP2	MN420907	*Paracoccus versutus strain NLB7 (97.9%), Paracoccus sp. strain CM1 (97.9%)*	*Paracoccus versutus strain ATCC 25364 (97.9%)*
3PUF_VP3	MN420908	*Acidithiobacillus thiooxidans strain S30A4 (99.8%)*	*Acidithiobacillus thiooxidans ATCC 19377 (99.8%)*
4PUF_VP4	MN420909	*Acidithiobacillus thiooxidans strain S30A4 (99.2%)*	*Acidithiobacillus thiooxidans ATCC 19377 (96.9%)*
5PUF_VP5	MN420910	*Acidithiobacillus thiooxidans strain S30A4 (99.2%)*	*Acidithiobacillus thiooxidans ATCC 19377 (99.8%)*
6PUF_VP6	MN420911	*Acidithiobacillus thiooxidans strain S30A4 (99.6%)*	*Acidithiobacillus thiooxidans ATCC 19377 (99.6%)*
7PUF_VP7	MN420912	*Acidithiobacillus thiooxidans strain S30A4 (97.3%)*	*Acidithiobacillus thiooxidans ATCC 19377 (97.3%)*
8PUF_VP8	MN420913	*Acidithiobacillus thiooxidans strain S30A4 (99.2%)*	*Acidithiobacillus thiooxidans (99.2%)*
9PUF_VP9	MN420914	*Acidithiobacillus sp. strain V1 (99.2%)*	*Acidithiobacillus caldus strain KU (96.3%)*
10PG_VP10	MN420915	*Thiomonas sp. (99.2%)*	*Thiomonas arsenitoxydans (99.2%), Thiomonas intermedia (99.1%)*

Table S5. Closest related microorganisms to nucleotide sequences excised from DGGE profiles from PG-horizontal pilot-scale biofilter compared with the NCBI nucleotide GenBank database

DGGE band	GenBank accession numbers	Closest related microorganisms to nucleotide sequences excised from DGGE profiles compared with GenBank database	
		**Standard databases (nr etc.)**	**rRNA/ITS databases (16 S rRNA)**
1PG_HP1	MN420869	*Paracoccus versutus strain NLB7 (99.2%), Paracoccus sp. strain CM1 (98.2%)*	*Paracoccus versutus strain ATCC 25364 (99.2%)*
2PG_HP2	MN420870	*Acidithiobacillus thiooxidans strain S30A4 (99.7%)*	*Acidithiobacillus thiooxidans ATCC 19377 (99.7%)*
3PG_HP3	MN420871	*Metallibacterium schefflera (99.2%)*	*Metallibacterium schefflera (99.2%)*
4PG_HP4	MN420872	*Paracoccus versutus strain NLB7 (97.2%), Paracoccus sp. strain CM1 (97.2%)*	*Paracoccus versutus strain ATCC 25364 (97.2%)*
5PG_HP5	MN420873	*Acidithiobacillus thiooxidans strain S30A4 (99.8%)*	*Acidithiobacillus thiooxidans ATCC 19377 (99.8%)*
6PG_HP6	MN420874	*Acidithiobacillus thiooxidans strain S30A4 (99.4%)*	*Acidithiobacillus thiooxidans ATCC 19377 (99.4%)*
7PG_HP7	MN420875	*Uncultured bacterium clone AS3cl15 (KU249580) (98.5%), Acidithiobacillus thiooxidans strain S30A4 (98.3%)*	*Acidithiobacillus thiooxidans ATCC 19377 (98.3%)*
8PG_HP8	MN420876	*Thiomonas sp. (99.6%)*	*Thiomonas arsenitoxydans (99.6%), Thiomonas intermedia (99.1%)*
9PG_HP9	MN420877	*Uncultured bacterium clone AS3cl15 (KU249580) (98.1%), Acidithiobacillus thiooxidans strain S30A4 (97.0%)*	*Acidithiobacillus thiooxidans ATCC 19377 (98.0%)*
10PG_HP10	MN420878	*Acidithiobacillus thiooxidans strain S30A4 (99.6%)*	*Acidithiobacillus thiooxidans ATCC 19377 (99.6%)*
11PG_HP11	MN420879	*Acidithiobacillus thiooxidans strain S30A4 (99.6%)*	*Acidithiobacillus thiooxidans ATCC 19377 (99.6%)*
12PG_HP12	MN420880	*Acidithiobacillus sp. strain V1 (99.4%)*	*Acidithiobacillus caldus strain KU (96.4%)*

Table S6. Closest related microorganisms to nucleotide sequences excised from DGGE profiles from PUF-horizontal pilot-scale biofilter compared with the NCBI nucleotide GenBank database

**DGGE band**	**GenBank accession numbers**	**Closest related microorganisms to nucleotide sequences excised from DGGE profiles compared with GenBank database**	
		**Standard databases (nr etc.)**	**rRNA/ITS databases (16 S rRNA)**
1PUF_HP1	MN420896	*Paracoccus versutus strain NLB7 (99.8%), Paracoccus sp. strain CM1 (99.8%)*	*Paracoccus versutus strain ATCC 25364 (99.8%)*
2PUF_HP2	MN420897	*Paracoccus versutus strain NLB7 (99.6%), Paracoccus sp. strain CM1 (99.6%)*	*Paracoccus versutus strain ATCC 25364 (99.6%)*
3PUF_HP3	MN420898	*Acidithiobacillus thiooxidans strain S30A4 (99.8%)*	*Acidithiobacillus thiooxidans ATCC 19377 (99.8%)*
4PUF_HP4	MN420899	*Acidithiobacillus thiooxidans strain S30A4 (99.8%)*	*Acidithiobacillus thiooxidans ATCC 19377 (99.8%)*
5PUF_HP5	MN420900	*Acidithiobacillus thiooxidans strain S30A4 (99.8%)*	*Acidithiobacillus thiooxidans ATCC 19377 (99.8%)*
6PUF_HP6	MN420901	*Acidithiobacillus thiooxidans strain S30A4 (99.8%)*	*Acidithiobacillus thiooxidans ATCC 19377 (99.8%)*
7PUF_HP7	MN420902	*Acidithiobacillus thiooxidans strain S30A4 (99.8%)*	*Acidithiobacillus thiooxidans ATCC 19377 (99.8%)*
8PUF_HP8	MN420903	*Acidithiobacillus thiooxidans strain S30A4 (99.8%)*	*Acidithiobacillus thiooxidans ATCC 19377 (99.8%)*
9PUF_HP9	MN420904	*Thiomonas intermedia (99.8%)*	*Thiomonas intermedia (99.8%)*
10PG_HP10	MN420905	*Acidithiobacillus thiooxidans strain S30A4 (97.3%)*	*Acidithiobacillus thiooxidans ATCC 19377 (97.7%)*

Table S7. The statistical analysis was performed using one-way analysis of variance (ANOVA) and Duncan's multiple range tests.

Packing materials	pH	Number of *P. versutus* CM1 on packing materials (log cfu/g)*				
		Days				
		1	2	3	4	5
PG	**4**	$9.57^{c} \pm 0.09$	$9.37^{b} \pm 0.03$	$9.41^{c} \pm 0.02$	$9.14^{b} \pm 0.02$	$8.93^{c} \pm 0.08$
	**5**	$9.73^{b} \pm 0.04$	$9.16^{c} \pm 0.02$	$9.62^{b} \pm 0.03$	$9.13^{b} \pm 0.03$	$9.20^{b} \pm 0.02$
	**7**	$9.96^{a} \pm 0.03$	$9.55^{a} \pm 0.07$	$9.75^{a} \pm 0.02$	$9.67^{a} \pm 0.13$	$9.85^{a} \pm 0.03$
	**12**	$8.38^{d} \pm 0.02$	$5.87^{d} \pm 0.03$	$5.17^{d} \pm 0.06$	$5.24^{c} \pm 0.02$	$4.95^{d} \pm 0.03$
PUF	**4**	$5.02^{c} \pm 0.73$	$4.11^{c} \pm 0.55$	$4.12^{c} \pm 0.55$	$3.32^{c} \pm 0.21$	$3.15^{c} \pm 0.61$
	**5**	$9.73^{b} \pm 0.02$	$9.66^{ba} \pm 0.06$	$8.93^{b} \pm 0.02$	$9.10^{b} \pm 0.14$	$7.81^{b} \pm 0.10$
	**7**	$10.64^{a} \pm 0.49$	$10.13^{a} \pm 0.02$	$10.92^{a} \pm 0.63$	$11.62^{a} \pm 0.07$	$11.27^{a} \pm 0.03$
	**12**	$9.99^{ab} \pm 0.03$	$9.39^{b} \pm 0.05$	$9.15^{b} \pm 0.02$	$9.28^{b} \pm 0.08$	$10.22^{b} \pm 0.03$

*All results with $p < 0.05$ are considered to be significantly different from each other. The data from different pH values for each day was compared together

Table S8. The statistical analysis was performed using one-way analysis of variance (ANOVA) and Duncan's multiple range tests.

Packing materials	Temp (°C)	Number of *P. versutus* CM1 on packing materials (log cfu/g)*				
		Days				
		1	2	3	4	5
PG	**25**	$10.61^{a} \pm 0.13$	$9.65^{a} \pm 0.02$	$9.79^{a} \pm 0.07$	$10.46^{a} \pm 0.01$	$9.76^{b} \pm 0.06$
	**30**	$9.99^{c} \pm 0.02$	$9.76^{a} \pm 0.13$	$9.73^{a} \pm 0.01$	$10.23^{ab} \pm 0.02$	$9.65^{c} \pm 0.04$
	**35**	$10.23^{b} \pm 0.15$	$9.77^{a} \pm 0.04$	$10.00^{a} \pm 0.43$	$9.98^{b} \pm 0.25$	$10.29^{a} \pm 0.55$
	**40**	$9.81^{c} \pm 0.09$	$9.73^{a} \pm 0.09$	$9.27^{b} \pm 0.06$	$9.14^{b} \pm 0.06$	$9.62^{b} \pm 0.26$
	**45**	$9.08^{d} \pm 0.07$	$8.13^{b} \pm 0.07$	$8.17^{c} \pm 0.02$	$8.26^{c} \pm 0.16$	$8.37^{d} \pm 0.09$
PUF	**25**	$11.78^{b} \pm 0.11$	$11.52^{c} \pm 0.02$	$11.81^{b} \pm 0.03$	$11.69^{b} \pm 0.04$	$11.36^{b} \pm 0.17$
	**30**	$11.52^{c} \pm 0.05$	$12.07^{a} \pm 0.09$	$12.08^{a} \pm 0.03$	$11.51^{c} \pm 0.09$	$11.50^{b} \pm 0.12$
	**35**	$12.73^{a} \pm 0.07$	$12.13^{a} \pm 0.05$	$12.13^{a} \pm 0.02$	$12.46^{a} \pm 0.05$	$12.65^{a} \pm 0.08$
	**40**	$11.46^{c} \pm 0.08$	$11.73^{b} \pm 0.07$	$10.98^{c} \pm 0.03$	$11.34^{d} \pm 0.01$	$11.16^{c} \pm 0.06$
	**45**	$11.21^{d} \pm 0.20$	$10.24^{d} \pm 0.05$	$9.87^{d} \pm 0.08$	$10.29^{e} \pm 0.02$	$10. 41^{d} \pm 0.06$

*All results with $p < 0.05$ are considered to be significantly different from each other. The data from different temperatures for each day was compared together.

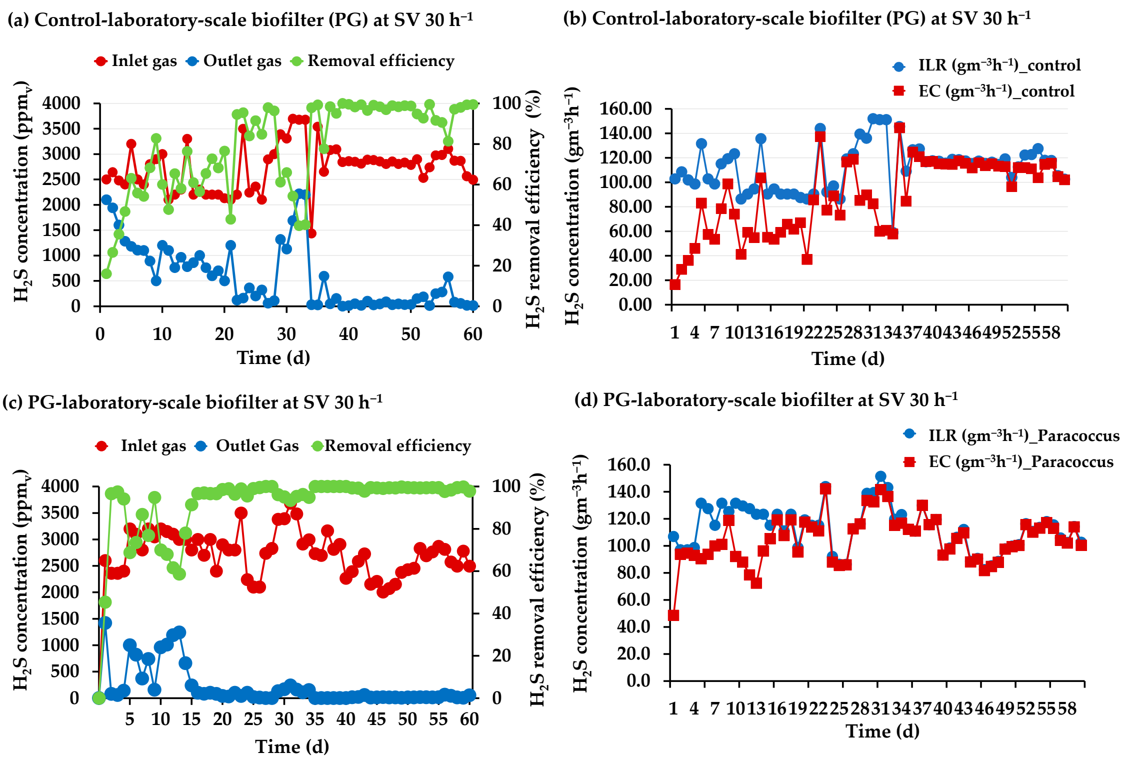

Figure S1. Comparison of biofilter performances between the PG laboratory-scale control biofilter without the immobilization of *Paracoccus versutus* CM1 and the PG laboratory-scale biofilter with *P. versutus* CM1.

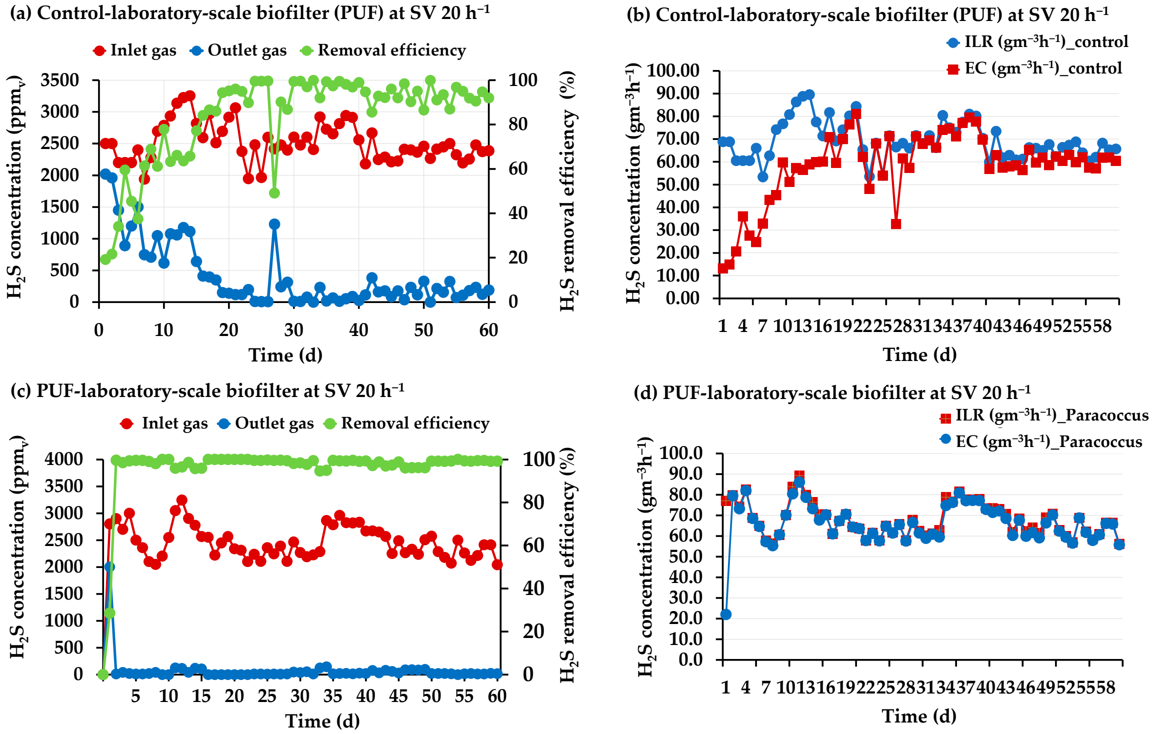

Figure S2. Comparison of biofilter performances between the PUF laboratory-scale control biofilter without the immobilization of *Paracoccus versutus* CM1 and the PUF laboratory-scale biofilter with *P. versutus* CM1

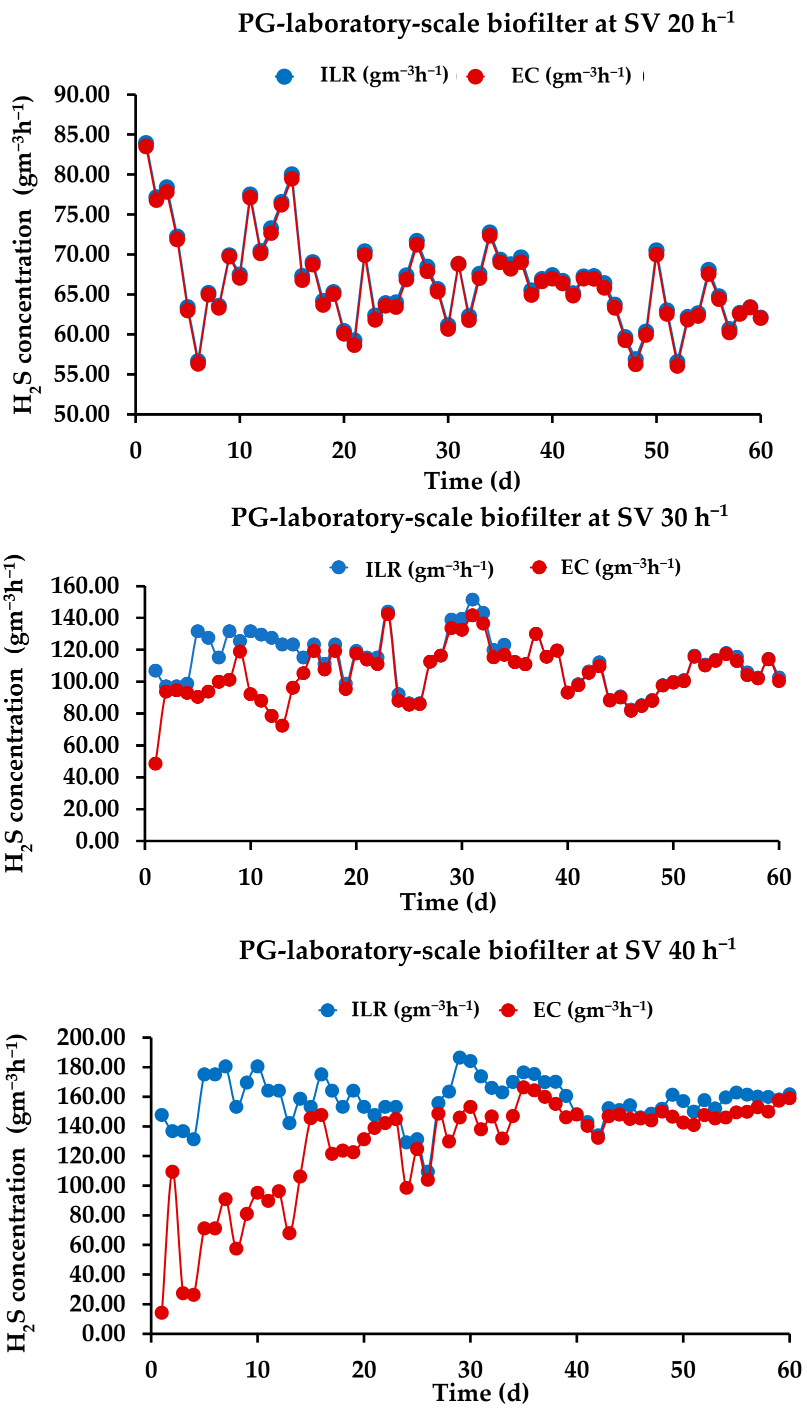

Figure S3. Laboratory-scale H_2S elimination using various SVs in PG biofilters

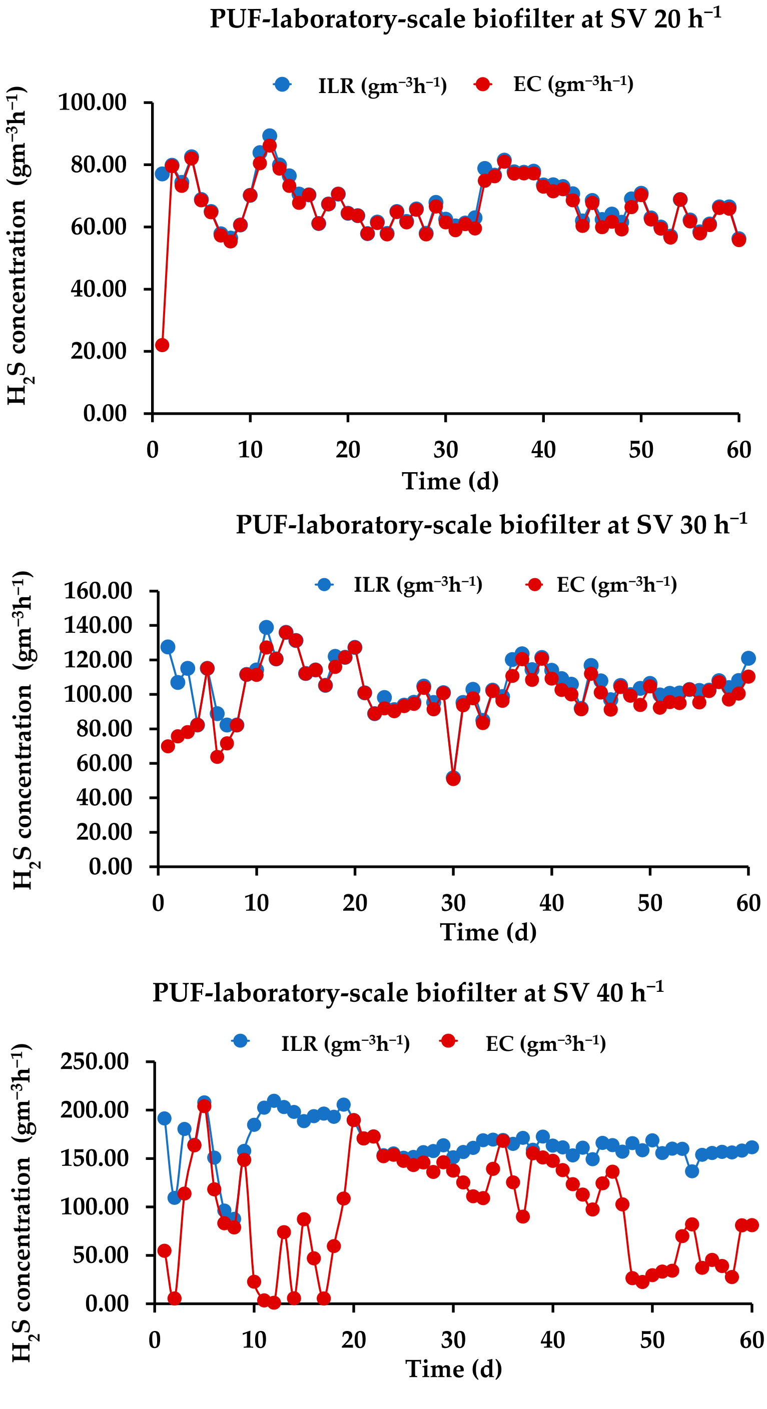

Figure S4. Laboratory-scale H_2S elimination using various SVs in PUF biofilters

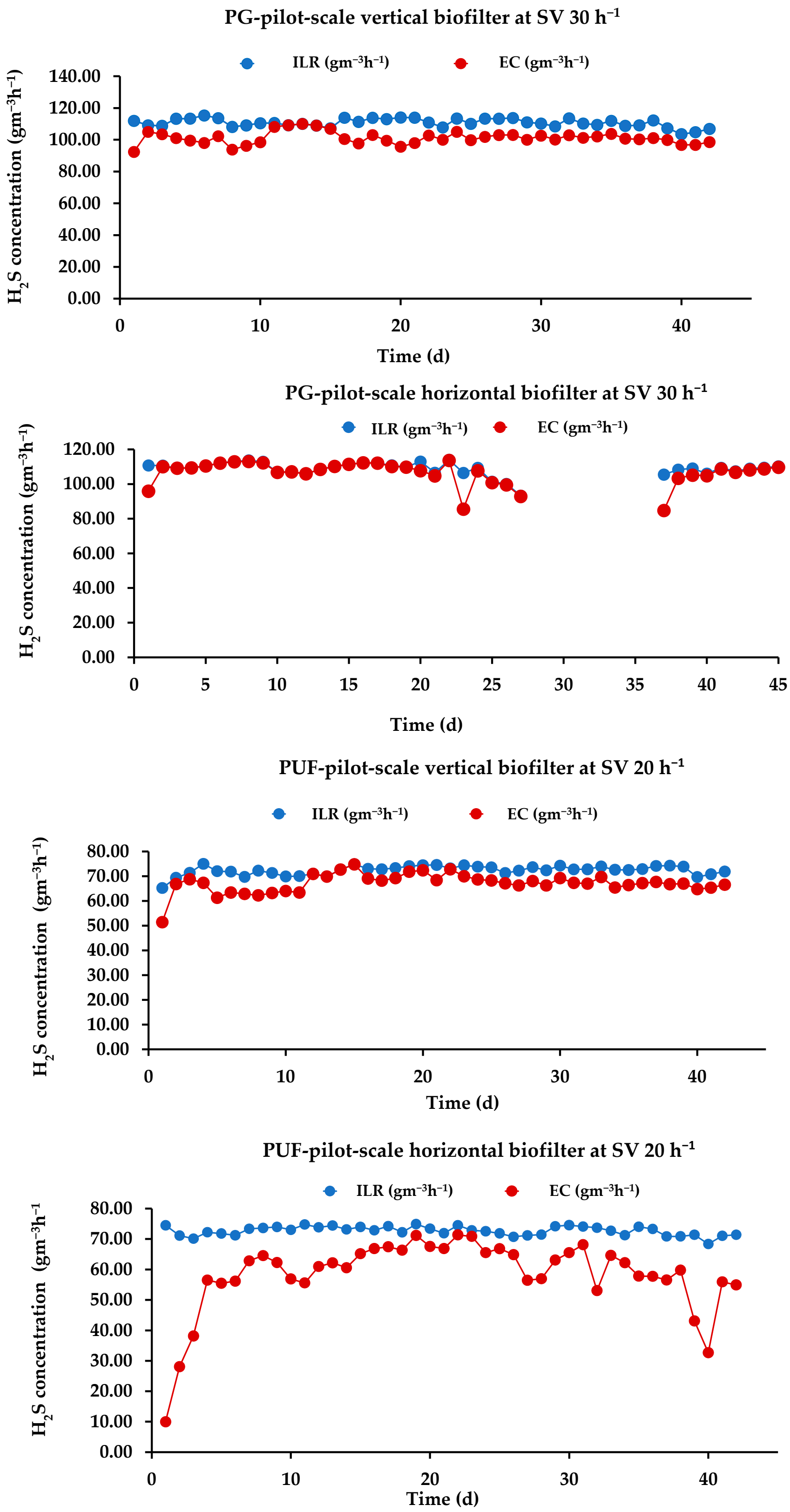

Figure S5. Pilot-scale H_2S elimination in vertical and horizontal PG and PUF biofilters

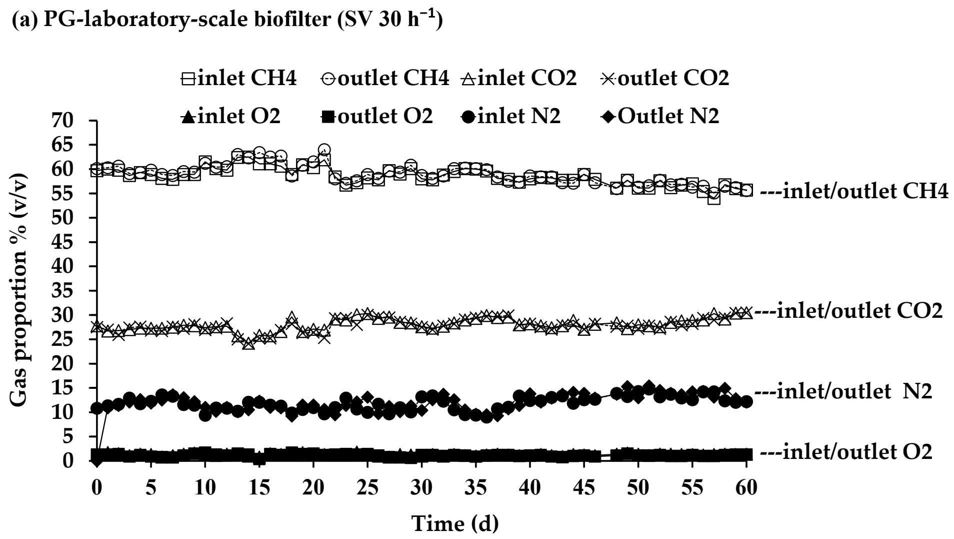

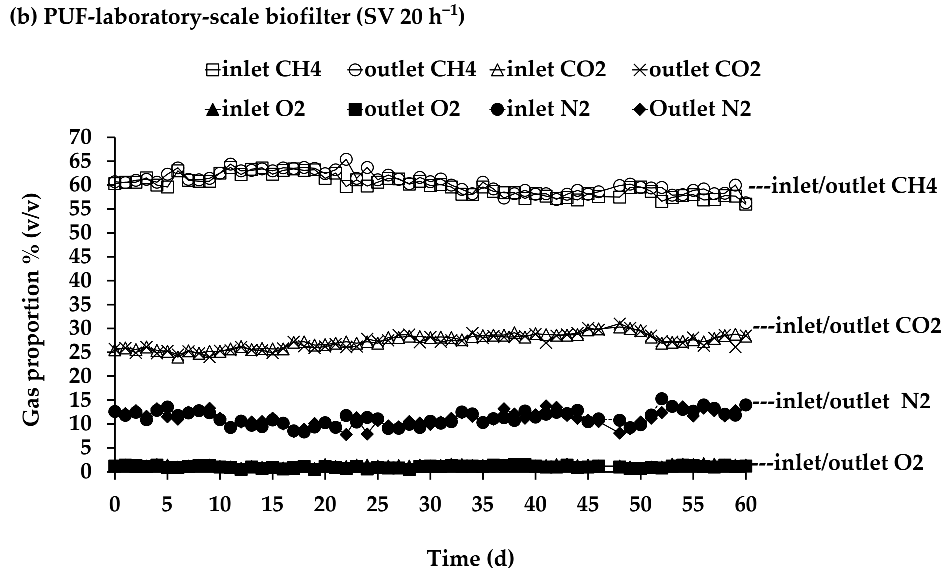

Figure S6. Biogas compositions in the laboratory-scale biofilters packed with immobilized *P. versutus* CM1 cells. (a) PG biofilter and (b) PUF biofilter. The biofiltration was operated at the SVs of 30 and 20 h^{-1}, respectively.

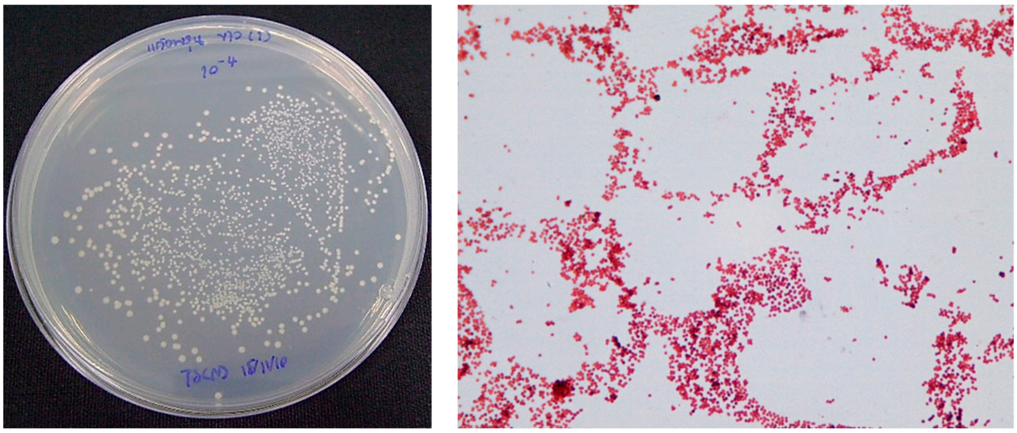

Figure S7. Colonies and cells of *P.versutus* CM1 on TSA medium collected from packing materials during the operation.

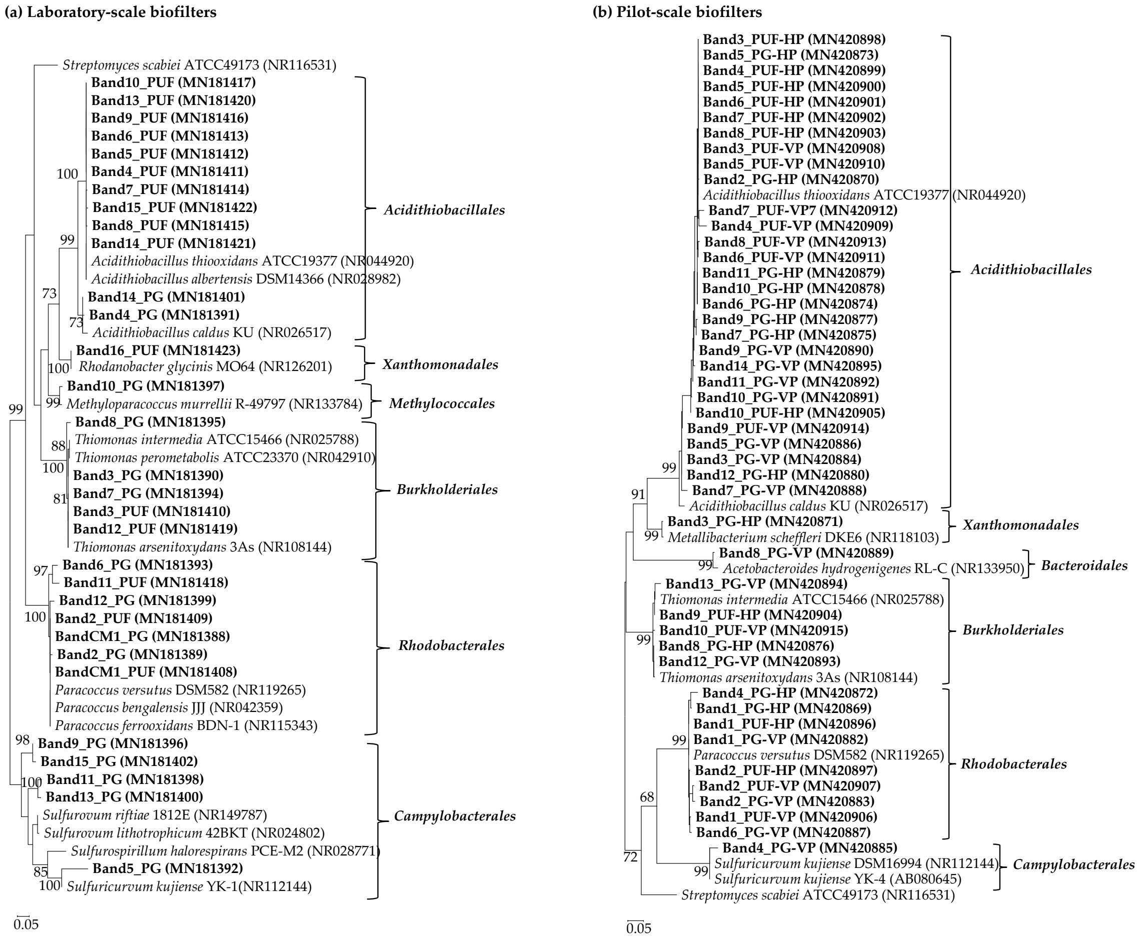

Figure S8. The phylogenetic tree constructed from partial 16S rRNA sequences of bacteria indicates the major bacterial taxa present in the PG and PUF of (a) laboratory-scale biofilters, and (b) pilot-scale biofilters using a neighbor-joining algorithm with 1,000 bootstrapping.
